# Autophagy in Pituitary Neuroendocrine Tumors (PitNETs): Molecular Mechanisms, Secretory Homeostasis, Therapeutic Resistance, and Translational Perspectives

**DOI:** 10.3390/ijms27209195

**Published:** 2026-10-16

**Authors:** Ida Rizzuto, Ludovica Pepe, Giuseppe Broggi, Rosario Caltabiano, Vincenzo Fiorentino, Antonio Ieni

**Affiliations:** 1 Anatomic Pathology Unit, Department of Human Pathology in Adult and Developmental Age “Gaetano Barresi”, University of Messina, 98125 Messina, Italy; ida.rizzuto@studenti.unime.it (I.R.); ludopepe97@gmail.com (L.P.); 2 PhD Program in Translational Molecular Medicine and Surgery, Department of Biomedical, Dental, Morphological and Functional Imaging Sciences, University of Messina, 98125 Messina, Italy; 3 Department of Medical and Surgical Sciences and Advanced Technologies “G.F. Ingrassia”, Anatomic Pathology, University of Catania, 95123 Catania, Italy; giuseppe.broggi@gmail.com (G.B.); rosario.caltabiano@unict.it (R.C.)

**Keywords:** pituitary neuroendocrine tumor, PitNET, autophagy, autophagic flux, mitophagy, crinophagy, drug resistance, dopamine agonist

## Abstract

Pituitary neuroendocrine tumors (PitNETs) are usually indolent but may show invasive growth, recurrence, hormonal hypersecretion, and treatment resistance. Autophagy intersects with nutrient sensing, lysosomal homeostasis, mitochondrial quality control, and secretory-granule turnover, but its biological effects in PitNETs are context-dependent. This narrative review synthesizes PitNET-specific and mechanistically relevant evidence on macroautophagy, mitophagy, crinophagy, non-coding RNA and epigenetic regulation, treatment resistance, and pharmacological modulation, emphasizing the distinction between static autophagy markers and dynamic autophagic flux. Evidence is interpreted across human tissue, primary human cells, pituitary tumor cell lines, animal models, and clinical observations, while preserving clear separation between human observations and mechanistic evidence derived from experimental models. Across experimental systems, autophagy activation is associated with antitumor effects in selected settings, whereas completed basal flux may support stress adaptation and late-stage or selective-autophagy inhibition may enhance cytotoxicity. Biological outcome therefore depends on tumor lineage, treatment, the pathway stage affected, and whether flux is completed. Recent studies extend this framework to selective mitophagy, ncRNA/exosomal resistance networks, epitranscriptomic regulation, somatostatin-analogue resistance, and tumor-microenvironmental signaling. Clinical translation remains limited, and no autophagy-modulating strategy is validated for routine PitNET management. Future studies should integrate dynamic flux measurements, lineage-specific biomarkers, pharmacodynamic target engagement, and prospective clinical endpoints.

## 1. Introduction: PitNET Clinical Context and Cellular Homeostasis

Pituitary neuroendocrine tumors (PitNETs), historically termed pituitary adenomas, arise from hormone-producing cells of the anterior pituitary. The 2022 World Health Organization classification favors the term PitNET while acknowledging the established adenoma terminology [1,2]. Most PitNETs are indolent, but a clinically important minority show invasive growth, recurrence, or, rarely, metastatic behavior [2,3]. A widely cited systematic review estimated an overall prevalence of 16.7% by combining autopsy and radiological series (14.4% and 22.5%, respectively); this estimate is driven largely by incidentally detected lesions and should not be interpreted as the prevalence of clinically manifest disease [4]. Population-based studies of clinically recognized pituitary tumors report a substantially lower but still important burden: a Belgian cross-sectional study found 94 clinically relevant cases per 100,000 inhabitants [5], an Icelandic nationwide series reported a prevalence of 115.6 per 100,000 and a standardized incidence rate of 5.8 per 100,000 person-years by 2012 [6], and a 30-year population-based analysis from Olmsted County reported a mean standardized incidence of 10.1 per 100,000 person-years and a final point prevalence of 175.1 per 100,000 [7]. Differences in case ascertainment, imaging use, diagnostic criteria, and calendar period should therefore be considered when comparing epidemiological estimates.

Invasive PitNETs may extend toward the optic chiasm, cavernous sinuses, and sphenoid bone, complicating surgical management and contributing to neurological morbidity [2,8]. Clinical manifestations arise from mass effect, including visual field impairment and headache, and/or endocrine dysfunction caused by autonomous hormone secretion or compression of normal pituitary tissue. Hypersecretory syndromes include acromegaly, Cushing disease, and hyperprolactinemia and may cause substantial metabolic, cardiovascular, skeletal, and neuropsychiatric morbidity. Despite advances in transsphenoidal surgery, radiotherapy, dopamine agonists, somatostatin receptor ligands, and other targeted medical treatments, invasive, recurrent, and treatment-refractory PitNETs remain challenging [2,9].

Altered cellular homeostasis and stress-response pathways are increasingly recognized as relevant to tumor biology, including PitNET biology. Autophagy is a conserved lysosome-dependent process that degrades and recycles damaged organelles, protein aggregates, and other cytoplasmic constituents, thereby contributing to energetic and proteostatic homeostasis [10,11]. Autophagy is dynamically regulated by nutrient availability, hypoxia, oxidative stress, and pharmacological injury, conditions that may also occur within tumor microenvironments [10,12].

In cancer, autophagy has a context-dependent dual role. Basal autophagy can limit the accumulation of damaged organelles and genomic stress during tumor initiation, whereas established tumors may use autophagy to tolerate metabolic and therapeutic stress [10,13,14,15,16,17]. Whether PitNETs undergo a fixed temporal switch from tumor-suppressive to tumor-promoting autophagy has not been demonstrated; available PitNET-specific studies instead indicate substantial heterogeneity according to tumor subtype, experimental context, and treatment exposure [9,18].

Pituitary endocrine cells also use specialized lysosomal quality-control pathways. Crinophagy directly targets secretory granules for lysosomal degradation and is particularly relevant to endocrine secretory homeostasis, whereas mitophagy selectively removes damaged mitochondria [19,20,21,22]. Evidence for their roles in human PitNET progression and treatment response varies substantially and should be distinguished from findings obtained in other endocrine tissues or experimental systems; direct human PitNET evidence remains particularly limited for crinophagy.

Two dedicated reviews published in 2025 summarized the emerging relationship between autophagy and pituitary tumors [9,18]. The present review adds a distinct evidence-stratified synthesis of PitNET-specific mechanistic and translational literature through 21 September 2026. In addition to recent work on epitranscriptomic and ncRNA regulation, selective mitophagy, dopamine- and somatostatin-analogue resistance, and tumor-microenvironmental signaling, we explicitly distinguish evidence from human PitNET tissue or primary cultures, pituitary tumor cell lines, in vivo pituitary models, and non-pituitary mechanistic contexts. We also grade the strength of autophagy assessment by separating static LC3/p62 or ultrastructural observations from methods that can interrogate dynamic flux. This model-and-method hierarchy is intended to clarify which findings are directly PitNET-specific, which remain mechanistic hypotheses, and which are sufficiently mature to inform translational study design.

## 2. Literature Search and Evidence Selection

Because the aim was interpretive rather than systematic, a targeted narrative literature search was used. PubMed/MEDLINE was searched and re-run through 21 September 2026 using combinations of the terms “pituitary adenoma”, “pituitary neuroendocrine tumor”, and “PitNET” with “autophagy”, “macroautophagy”, “mitophagy”, “crinophagy”, “lysosome”, “mTOR”, “AMPK”, “drug resistance”, “dopamine agonist”, “somatostatin”, “somatostatin analogue”, “octreotide”, “pasireotide”, “endoplasmic reticulum stress”, “microRNA”, “miRNA”, “lncRNA”, “circRNA”, “exosome”, “tumor microenvironment”, “immune microenvironment”, “STING”, “m6A”, “N6-methyladenosine”, “RNA methylation”, “METTL14”, “epitranscriptomics”, “TP53”, “USP8”, “USP48”, “BRAF”, “GNAS”, “AIP”, and “temozolomide”. The core Boolean structure was (“pituitary adenoma” OR “pituitary neuroendocrine tumor” OR PitNET) AND (autophagy OR macroautophagy OR mitophagy OR crinophagy OR lysosome OR mTOR OR AMPK OR “drug resistance” OR temozolomide OR microRNA OR lncRNA OR circRNA OR exosome OR “tumor microenvironment” OR STING OR TP53 OR USP8 OR USP48 OR BRAF OR GNAS OR AIP), with pathway- and treatment-specific terms added as listed above. Reference lists of recent PitNET-autophagy reviews and of included primary studies were screened for additional mechanistic reports. ClinicalTrials.gov was checked on 21 September 2026 to verify the status and posted results of NCT03400865. Priority was given to original studies using human PitNET tissue, primary human PitNET cultures, pituitary tumor cell lines, or in vivo pituitary tumor models. Non-pituitary studies were retained only when required to define conserved autophagy mechanisms not directly established in PitNETs. Evidence was interpreted hierarchically according to proximity to human PitNET biology, and static LC3 or p62 measurements were not treated as equivalent to dynamic autophagic-flux assessment. The updated search also identified newly available human PitNET genomic and immune-microenvironment studies, which were incorporated as genomic or microenvironmental context without treating them as direct evidence of autophagic flux. This was a narrative review; no PRISMA-based study selection, formal risk-of-bias assessment, or quantitative synthesis was performed. The principal limitations are the targeted, non-systematic literature selection, the predominance of preclinical evidence, heterogeneity across models, and the frequent reliance of available studies on static autophagy markers rather than dynamic-flux measurements.

## 3. Core Molecular Machinery of Autophagy: Conserved Principles and PitNET-Relevant Evidence

Autophagy in pituitary cells is governed by the conserved autophagy-related (ATG) machinery and by nutrient- and stress-sensing signaling pathways. The molecular sequence described below is therefore primarily the canonical mammalian macroautophagy framework, not a claim that every component is uniquely or uniformly dysregulated in PitNETs. PitNET-specific relevance is strongest where human tumor tissue, primary cultures, or dedicated pituitary models have directly interrogated pathway components; these levels of evidence are identified explicitly throughout the review [18,23].

### 3.1. Macroautophagy: Canonical Pathways and Structural Progression

Macroautophagy is the best-characterized form of autophagy [12,24,25]. It begins with the formation of a phagophore that expands into a double-membrane autophagosome, sequestering cytoplasmic cargo before fusion with lysosomes [23]. Studies in mammalian systems indicate that early autophagy initiation can involve liquid–liquid phase separation (LLPS), which concentrates signaling and membrane-remodeling factors at defined subcellular sites, including endoplasmic-reticulum-associated domains [12,23]. This biophysical mechanism has not yet been specifically established as a defining feature of PitNET autophagy.

A central regulator of initiation is the Unc-51-like autophagy activating kinase 1 (ULK1) complex. Following appropriate metabolic signaling, ULK1 promotes phagophore initiation and recruitment of the class III phosphatidylinositol 3-kinase complex containing VPS34, VPS15, and Beclin-1 (BECN1) [26,27]. Production of phosphatidylinositol 3-phosphate at the nascent autophagic membrane recruits downstream effectors required for membrane expansion and cargo sequestration.

Autophagosome elongation and closure involve two ubiquitin-like conjugation systems, including the ATG12-ATG5-ATG16L1 complex and the LC3 conjugation pathway [26]. Pro-LC3 is cleaved by ATG4 to generate cytosolic LC3-I and is subsequently conjugated to phosphatidylethanolamine through ATG7- and ATG3-dependent reactions to form membrane-associated LC3-II. LC3-II is widely used as a marker of autophagosome abundance; however, a static increase in LC3-II does not by itself establish increased autophagic flux because LC3-II can accumulate either when autophagosome formation increases or when downstream degradation is impaired, as also illustrated by tissue-level profiling of autophagy-related proteins in other human tumor types [28,29]. Assessment of flux therefore requires dynamic turnover approaches, lysosomal inhibition and/or complementary imaging and cargo-clearance readouts [26,30]. The interpretive strengths and limitations of the principal assays used in PitNET studies are summarized in Table 1.

After closure, mature autophagosomes fuse with lysosomes to form autolysosomes, in which acidic hydrolases degrade the inner autophagosomal membrane and its cargo [26]. The resulting amino acids, fatty acids, sugars, and other metabolites can be returned to the cytosol and reused. This recycling function supports cellular homeostasis and, under some oncogenic contexts, may also help established tumor cells tolerate nutrient or treatment stress [12,26]. The core stages and molecular components of macroautophagy are summarized in Table 2 as a framework for interpreting PitNET-specific findings.

### 3.2. Ultrastructural Correlates of Autophagy in PitNETs

Transmission electron microscopy (TEM) provides structural evidence that autophagy-related compartments occur in PitNETs, while remaining insufficient by itself to establish dynamic flux. In the study by Cecenarro et al., 20 of 33 functioning PitNETs showed cytoplasmic vacuolization and membrane-limited vesicular structures containing cytoplasmic material, secretory-granule remnants and, in some fields, organellar cargo; these structures were morphologically distinguishable from electron-dense conventional lysosomes [31]. Similar double-membrane vesicles at different degradative stages were documented in experimental pituitary tumors [31]. These observations strengthen the morphological basis for macroautophagy in pituitary tumors, but TEM is a static snapshot and should be integrated with turnover assays, tandem fluorescent reporters, or lysosomal-function measurements when the aim is to infer flux [30,31].

## 4. Autophagy-Regulating Signaling in PitNETs: Kinase Networks and Genomic Context

Basal autophagy and stress-induced autophagy are regulated by interconnected kinase networks rather than by a single linear pathway. In PitNETs, several of these signaling axes, particularly PI3K/AKT/mTOR and RAF/MEK/ERK, have been implicated in tumor growth, invasiveness, and treatment response, although their relationship with autophagic flux is not identical across tumor subtypes [32,33].

### 4.1. The PI3K/AKT/mTOR Axis: A Major Negative Regulator

Mechanistic target of rapamycin complex 1 (mTORC1) is a major nutrient sensor and negative regulator of macroautophagy [32]. Under nutrient-replete conditions, active mTORC1 phosphorylates components of the ULK1 complex and restrains autophagy initiation, while simultaneously promoting anabolic processes such as protein synthesis. When mTORC1 activity falls, ULK1 can become activated and initiate the autophagic program [26,32].

The PI3K/AKT/mTOR pathway is frequently activated in PitNETs and has been investigated as a contributor to proliferation and aggressive behavior [32,33]. PTEN counteracts PI3K signaling by dephosphorylating PIP3 and thereby limiting AKT activation. Reduced PTEN expression has been associated with invasive pituitary adenomas in a tissue-based study, but the available evidence does not justify describing PTEN loss as a universal or defining molecular event of recurrent or aggressive PitNETs [34]. Functionally, attenuation of PI3K/AKT/mTOR signaling can relieve mTORC1-mediated suppression of autophagy, although the downstream biological effect remains context-dependent.

### 4.2. The AMPK Pathway: Energy Sensing and Positive Regulation

AMP-activated protein kinase (AMPK) acts as an intracellular energy sensor and can promote autophagy during energy depletion, hypoxia, or glucose limitation [35]. AMPK can inhibit mTORC1, including through TSC2, and can also directly phosphorylate ULK1 at activating sites. Direct human PitNET data show increased phosphorylated AMPK (Thr172) in sporadic somatotroph tumors and in GH-secreting tumors from patients with germline *AIP* variants compared with normal pituitary tissue [36]. This observation supports activation of the AMPK pathway in at least some somatotroph PitNETs, but it does not by itself establish increased autophagic flux or prove that chronic AMPK-driven autophagy is a universal survival mechanism in hypoxic macroadenoma cores [18,35,36].

Additional PitNET-specific data link mitochondrial dysfunction to AMPK-ULK1 signaling. In a 2022 study of 20 human pituitary adenomas (10 low- and 10 high-proliferation-index tumors) and experimental models, Drp1 expression was higher in the low-proliferation group. In GH3 cells, Drp1 overexpression increased mitochondrial ROS, impaired oxidative phosphorylation and ATP production, activated AMPK-ULK1 signaling, increased LC3 and reduced p62. Pharmacological autophagy inhibition with 3-MA increased apoptosis. Drp1 overexpression also suppressed tumor growth in vivo. These findings support a Drp1-mitochondrial dysfunction-AMPK/ULK1 axis in which the induced autophagy-related response may be cytoprotective rather than uniformly antitumor [37].

### 4.3. Signaling Interactions and Reciprocal Feedback Loops

Autophagy-regulating signaling is characterized by reciprocal feedback. For example, mTORC1-S6K signaling can inhibit upstream insulin-receptor-substrate signaling, thereby constraining PI3K activation. Such feedback is well established in growth-factor signaling and may influence the cellular response to pathway-directed drugs. Its specific quantitative contribution to basal autophagic flux in individual PitNET subtypes has not been established [32,33].

Crosstalk between RAF/MEK/ERK and PI3K/AKT/mTOR signaling has been described in PitNETs, including aggressive tumors [33]. Because both pathways influence proliferation, survival, metabolism, and autophagy-related signaling, compensatory pathway activation provides a biologically plausible mechanism of treatment adaptation. Nevertheless, the proposition that pathway inhibition invariably restores autophagic homeostasis through a parallel cascade should be regarded as a mechanistic hypothesis rather than a demonstrated universal response in PitNETs.

Defining which signaling alterations are present in a given PitNET, and whether they are accompanied by increased autophagosome formation or impaired downstream degradation, is therefore important for rational interpretation of autophagy-directed therapeutic strategies.

### 4.4. Oncogenic Alterations and Autophagy: Established Intersections and Evidence Gaps

The relationship between PitNET genomic alterations and autophagy requires careful separation of established tumor genetics from autophagy-specific evidence. *TP53* is a major regulator of autophagy in general cancer biology: cytoplasmic p53 can suppress basal autophagy [38], whereas nuclear p53 can promote autophagy through transcriptional targets such as damage-regulated autophagy modulator (*DRAM*) [39]. Pathogenic *TP53* alterations are uncommon in unselected PitNETs but enriched in selected corticotroph and clinically aggressive cohorts. Whole-exome sequencing of a corticotroph series enriched for tumors at risk of aggressive behavior identified recurrent, mutually exclusive *USP8* and *TP53* alterations [40]; in a separate series of 86 functional corticotroph tumors, pathogenic *TP53* variants were detected in 9 tumors, all macroadenomas and *USP8* wild type, representing 24% of invasive tumors in that selected cohort [41]. In a broader cohort, *TP53*-mutant PitNETs showed a higher fraction of genome-wide loss of heterozygosity (LOH), whereas LOH itself—not *TP53* status alone—emerged as the strongest genomic predictor of aggressive, treatment-refractory behavior [42]. A 2026 integrated multi-omic study of 206 patients further identified lineage-specific molecular signatures of aggressiveness and associations with alterations including *TP53* and *CDKN2A*, while underscoring that genomic determinants differ across PitNET lineages [43].

These genomic observations are clinically relevant, but none demonstrates that a specific PitNET genotype produces a defined autophagic-flux phenotype. Other lineage-associated drivers and predisposition contexts include *GNAS* and occasional somatic *MEN1* alterations in the broader PitNET mutational landscape [44], recurrent *USP48* and *BRAF* alterations in *USP8*-wild-type corticotroph tumors [45], and germline *AIP* variants in a minority of patients, particularly younger patients with large tumors [46]. PTEN/PI3K pathway dysregulation intersects directly with the mTOR-autophagy axis [32,34], but even here pathway dysregulation should not be equated with measured flux. Mutation status should therefore currently be viewed as a biological context variable rather than as a stand-alone autophagy biomarker. Future studies should pair genomic classification with dynamic flux measurements in matched human tumors and treatment-response models.

## 5. Context-Dependent Roles of Autophagy in PitNET Biology

Autophagy can exert apparently opposing effects during cancer development. By limiting oxidative and proteotoxic damage, basal autophagy may suppress early tumorigenic events, whereas established neoplasms can use autophagy to survive metabolic, hypoxic, and treatment-related stress [13,14,15]. This conceptual framework is relevant to PitNETs, but a fixed stage-dependent switch has not been demonstrated in patients. Direct PitNET studies instead support a context-dependent relationship between autophagy, tumor size, invasive behavior, and pharmacological response [9,18,31,47].

### 5.1. Autophagy as a Potential Tumor-Suppressive Mechanism in Selected Contexts

Basal autophagy removes damaged mitochondria and misfolded proteins and can limit reactive oxygen species, proteotoxic stress, and chronic inflammatory signaling [13,14,15]. These functions can reduce conditions that favor genomic instability and transformation. In PitNETs, this tumor-suppressive concept is biologically plausible and is supported by experimental observations in which induction of autophagy can accompany reduced proliferation or invasion, but evidence directly reconstructing the earliest stages of human PitNET oncogenesis is necessarily limited [18,47].

Direct corticotroph-model evidence also supports an antitumor effect of autophagy induction in selected contexts. In AtT20/D16v-F2 cells, the BMP4-signaling agonist BMPSB4 activated SMAD1/5/9 and induced autophagy documented by transmission electron microscopy and tandem mRFP-EGFP-LC3 imaging; interference with the autophagic response attenuated the growth-inhibitory effect. In vivo, BMPSB4 reduced tumor growth and ACTH and corticosterone secretion, supporting a BMP4/SMAD-dependent autophagy-mediated antitumor mechanism in corticotroph PitNET models [48].

Autophagy and apoptosis are also molecularly interconnected. Beclin-1 forms part of the class III PI3K complex required for autophagy initiation, whereas anti-apoptotic BCL-2 family proteins can bind Beclin-1 and inhibit Beclin-1-dependent autophagy [49]. This interaction does not support a simple model in which Beclin-1 acts primarily by sequestering BCL-2/BCL-xL to release BAX/BAK. Instead, autophagic and apoptotic signaling share regulatory proteins whose functional consequences depend on cellular context and stress intensity [10,49].

### 5.2. Autophagy in Established Tumors and Models: Context-Dependent Stress Adaptation and Growth Regulation

In established solid tumors, autophagy can provide substrates for bioenergetic and biosynthetic pathways and can mitigate organelle damage during hypoxia, nutrient limitation, and therapy [12,13,14,15]. These principles offer a plausible framework for large PitNETs, particularly when tumor growth creates metabolic stress, but they do not establish that every macro-PitNET is dependent on autophagy for survival.

Direct human PitNET evidence is available. Cecenarro and colleagues evaluated functioning PitNETs using ultrastructural and molecular approaches and reported evidence of macroautophagy in approximately 60% of tumors, with differences in Beclin-1 and LC3 expression according to tumor size [31]. These findings support an association between autophagy-related markers and tumor size/phenotype but do not, by themselves, prove increased dynamic autophagic flux or a causal transition from microadenoma to an ‘autophagy-addicted’ macroadenoma. Complementing this observation, a 2024 cell-line study found that HES1/ITPR1-associated autophagy exerted anti-proliferative and anti-invasive effects, underscoring the context-dependent nature of autophagy in pituitary tumor models [47].

A 2026 study identified a further context-specific regulatory circuit involving SOX6, LAMP5, and NF-κB. LAMP5 was increased in pituitary adenoma samples, and *LAMP5* knockdown reduced autophagy, proliferation, migration, and xenograft growth in GH3 models; rapamycin partially rescued the proliferative phenotype. SOX6 acted as a negative transcriptional regulator of *LAMP5* and suppressed NF-κB-dependent autophagy [50]. Because autophagy assessment relied largely on autophagosome staining and static molecular readouts rather than comprehensive dynamic-flux assays, these findings are best interpreted as mechanistic evidence for a SOX6-LAMP5-autophagy growth axis rather than proof of a uniform increase in autophagic flux in human PitNETs.

A separate 2026 integrative study associated the N^6^-methyladenosine (m^6^A) writer-complex component METTL14 with invasive PitNET phenotypes using human bulk transcriptomic data and single-cell RNA sequencing. Functional experiments in the non-endocrine folliculo-stellate-like mouse pituitary TtT/GF line and the murine corticotroph AtT20 line showed that *METTL14* overexpression increased proliferation and invasion, enhanced insulin-like growth factor 2 (*IGF2*) expression through m^6^A modification, and activated PI3K/AKT signaling. Autophagy-related effects were inferred from increased LC3B-II/I, reduced p62, and increased ATG5 and Beclin-1, with reciprocal changes after *METTL14* knockdown. Because dynamic autophagic flux was not directly measured, these findings support a context-specific METTL14-IGF2/PI3K-AKT-associated autophagy mechanism rather than definitive evidence of increased flux in human PitNETs. The coexistence of PI3K/AKT activation with autophagy-related marker changes is non-canonical relative to the conventional PI3K/AKT/mTOR model and should therefore be interpreted cautiously in the absence of direct flux measurements [51].

Recent somatotroph-PitNET data introduce the tumor microenvironment as a modifier of autophagy. In human somatotroph PitNET tissue, cathepsin Z (CTSZ) co-localized with CD68-positive macrophages and correlated with the M2-like macrophage marker CD163. In GH3 cells, macrophage-conditioned medium increased proliferation, migration, invasion, and GH secretion while reducing autolysosome abundance and LC3-II expression, with transcriptomic and targeted analyses implicating TGF-β signaling. Recombinant CTSZ alone did not reproduce the proliferative or secretory phenotype, so CTSZ is best regarded as a macrophage-associated marker rather than as the demonstrated causal autophagy regulator. These findings support macrophage–tumor-microenvironment/autophagy interactions in experimental models but do not establish dynamic flux in human somatotroph PitNETs [52].

Two additional human studies strengthen the tumor-microenvironment context while also illustrating the need to separate association from autophagy measurement. In 48 PitNET samples, aggressive tumors showed greater T-lymphocyte and CD163-positive macrophage infiltration than non-aggressive tumors, and CD163 was associated with recurrence [53]. A separate multiplex-immunofluorescence study reported higher total macrophage infiltration and STING positivity in PitNETs than in normal pituitary tissue, with correlations between macrophage/STING measures and Ki-67 and exploratory differences according to *AIP* status [54]. Neither study directly measured autophagic flux; accordingly, these observations support an immune-microenvironmental context in which autophagy may be relevant, not direct evidence that autophagy is activated or suppressed in those human tumors.

A 2026 region-resolved multi-omics study provides an additional spatial human perspective. In 10 Knosp grade 4 PitNETs with paired saddle and cavernous-sinus specimens, proteomic enrichment analysis showed that proteins enriched in the saddle region were linked to autophagy, whereas cavernous-sinus-enriched proteins were associated mainly with MAPK/IGF1R signaling, proline transport, and extracellular-matrix programs [55]. These findings represent pathway-enrichment evidence rather than direct measurement of autophagic flux and do not indicate greater autophagy-related enrichment in the cavernous-sinus invasive compartment than in the matched saddle compartment.

Accordingly, PitNET autophagy should not be reduced to a single directional model. In human functioning PitNETs, ultrastructural and molecular observations support the presence of macroautophagy and associations with tumor phenotype, but do not establish a universal direction of dynamic flux [31]. In the HES1/ITPR1 model, autophagy induction was associated with anti-proliferative and anti-invasive effects [47]. By contrast, *TFE3* silencing or pharmacological inhibition of autophagic/lysosomal pathways suppressed proliferation and hormone production in pituitary tumor cell models, and chloroquine showed additive growth inhibition with temozolomide [56]. Cabergoline can induce autophagy while impairing late-stage degradative completion, producing autophagy-related cell death in selected models [57], whereas inhibition of mitophagy through the p300-H3K18-lactylation-WASH1 axis can increase mitochondrial ROS and potentiate dopamine-agonist cytotoxicity [58]. These apparently divergent results reflect different tumor lineages, initiating stimuli, autophagy stages, doses and time points rather than a single contradiction in the literature.

### 5.3. PitNET-Specific Biology Versus General Cancer-Autophagy Mechanisms

Several mechanisms reviewed here are shared with cancer biology broadly. mTOR-mediated repression of ULK1 and AMPK-dependent responses to energetic stress are conserved upstream regulators of autophagy [10,30], while p53–autophagy interactions are established in non-pituitary systems [38]. Likewise, lysosomal dependence under treatment stress and the possibility that either excessive autophagy or late-stage blockade can become cytotoxic belong to the broader cancer-autophagy framework [12,13,14,15,59]. Their presence in cancer biology should not be mistaken for PitNET specificity. PitNET-specific relevance emerges when these mechanisms intersect with endocrine-lineage features and directly studied resistance circuits: *miR-93*/ATG7 in dopamine-agonist resistance [60], exosomal *circOMA1*/DRD2 signaling [61], Connexin 36-mediated intercellular stress transmission during somatostatin-analogue treatment [62], and p300-regulated mitophagy during dopamine-agonist exposure [58]. Crinophagy is especially informative in this distinction: it is firmly grounded in pituitary/endocrine secretory physiology, yet direct evidence that tumor-specific crinophagic flux drives human PitNET progression remains limited [22,63]. This separation between conserved cancer mechanisms and PitNET-specific validation is central to interpreting translational claims.

## 6. Selective Lysosomal Quality-Control Pathways: Mitophagy, Crinophagy, and Secretory Homeostasis

### 6.1. Mitophagy: Mitochondrial Quality Control and Treatment Response

Mitophagy is the selective autophagic removal of damaged or superfluous mitochondria and is essential for mitochondrial quality control [21,64]. Mitochondrial turnover is particularly relevant to endocrine cells because hormone synthesis and secretion impose substantial energetic demands. In PitNETs, however, direct studies of mitophagy have historically been scarce, and mechanistic conclusions derived from other tumor types should not automatically be transferred to pituitary tumors.

The GH-GHR-IGF1 axis influences systemic metabolism and mitochondrial function, including mitochondrial biogenesis, oxidative phosphorylation, and quality-control pathways [19]. These observations provide a physiological rationale for studying mitophagy in somatotroph cells, but they do not establish that GH hypersecretion by itself produces a defined mitophagy phenotype in human somatotroph PitNETs. Earlier models that portrayed defective mitophagy as a proven driver of oxidative mutagenesis in somatotropinomas therefore require qualification [19,21].

More direct PitNET evidence has recently emerged. A 2024 study integrating transcriptomic data from 32 postoperative pituitary tumors with quantitative proteomics from 19 tumors identified HSPD1 as a mitophagy-associated candidate linked to invasiveness [65]. HSPD1 expression was lower in invasive than in non-invasive tumors in the analyzed datasets, and HSPD1 overexpression reduced proliferation, migration, and invasion in GH3 cells. These findings connect mitochondrial quality-control biology with PitNET behavior, although they do not establish a single global direction of mitophagic flux in invasive tumors [65].

A 2026 study further demonstrated that mitophagy can influence dopamine-agonist responsiveness in prolactinoma models [58]. In human prolactinoma specimens, p300 expression was reduced in tumors with poor dopamine-agonist responsiveness and was inversely associated with dopamine-agonist dosage. Mechanistically, the authors linked p300-mediated histone H3K18 lactylation to increased NDUFS7 and WASH1 expression; WASH1 impaired p62-dependent recognition and clearance of damaged mitochondria, thereby suppressing mitophagy, sustaining mitochondrial reactive oxygen species, and enhancing dopamine-agonist-induced apoptosis. The p300 HAT-domain activator YF-2 also potentiated dopamine-agonist effects in cellular and xenograft models [58]. Thus, the human data support an association between p300 and treatment responsiveness, whereas the mitophagy mechanism and YF-2 sensitization remain preclinical. These data are important because they show that, in at least one treatment context, inhibition rather than activation of mitophagy can favor tumor-cell killing. Current evidence therefore supports a subtype- and treatment-dependent role for mitophagy in PitNETs rather than a simple model of progressively increasing mitophagy during tumor growth.

### 6.2. Crinophagy: Lysosomal Regulation of Secretory Granules

Crinophagy is a lysosomal pathway in which secretory granules are delivered directly to lysosomes for degradation without canonical autophagosome formation. It was originally described ultrastructurally in anterior pituitary cells and later studied in multiple secretory systems [22,63]. Because pituitary cells continuously synthesize, store, and release peptide hormones, crinophagy is physiologically relevant to secretory-granule homeostasis; however, its quantitative contribution to human PitNET behavior remains poorly defined.

Evidence for the detailed molecular machinery is uneven across species and tissues. Pituitary physiology supports a role for lysosomal disposal of secretory granules, whereas several specific tethering and fusion mechanisms have been established mainly in experimental endocrine systems rather than in human PitNETs [22,63]. Therefore, physiological plausibility should be separated from direct tumor evidence.

### 6.3. SNARE Proteins and the Mechanics of Secretory Fusion

Genetic studies in Drosophila secretory cells implicate the HOPS tethering complex, Rab-family GTPases, PLEKHM1-related machinery, lysosomal acidification, and SNARE proteins in crinophagic delivery [63,66]. These data provide a mechanistic framework, but they do not demonstrate that the identical molecular complex governs crinophagy in human PitNETs.

Similarly, Ykt6-Snap29-Syntaxin 13 and related VAMP-dependent complexes have been characterized in Drosophila crinophagy, whereas mammalian pancreatic beta cells use VAMP4 to route a subset of nascent insulin granules toward lysosomal degradation [66,67]. The beta-cell finding establishes a mammalian endocrine precedent, not a PitNET-specific mechanism.

### 6.4. Crinophagic Dysregulation and Targeted Secretion Inhibitors

In functioning PitNETs, altered secretory-granule turnover could plausibly influence hormone storage or release, but a causal chain in which defective crinophagy directly produces hypersecretion has not been demonstrated in patients [18,63]. Crinophagy should therefore be regarded as a relevant secretory-homeostasis process whose tumor-specific contribution remains unresolved.

Likewise, lysosomal degradation of secretory granules returns metabolites to the cytosolic pool, but the proposal that PitNETs deliberately increase crinophagy to use peptide hormones as a major metabolic fuel source has not been directly tested and should remain explicitly hypothetical [63].

Human pituitary studies provide more direct evidence for the secretory SNARE machinery than for tumor-specific crinophagic flux. VAMP1, VAMP2, and VAMP3 have been characterized in human somatotroph adenomas, motivating development of targeted secretion inhibitors (TSIs) derived from botulinum neurotoxin domains [68]. The experimental qGHRH-LH(N)/D construct was designed to target GHRH-receptor-expressing somatotroph cells and cleave VAMP proteins. Although catalytic cleavage was demonstrated in cell-free preparations, the construct did not significantly suppress GH secretion in cultured human somatotroph tumor cells [68]. Thus, TSIs remain an informative proof-of-concept rather than an established therapeutic strategy.

## 7. Epigenetic and Post-Transcriptional Control of Autophagy and Drug Response

Autophagy-related pathways in PitNETs are also influenced by post-transcriptional regulation. MicroRNAs (miRNAs), long non-coding RNAs (lncRNAs), and circular RNAs (circRNAs) can alter expression of autophagy-related genes and signaling components, providing mechanisms through which tumor cells may modify autophagic responses and drug sensitivity [69,70,71]. The strength of evidence is greatest for a limited number of experimentally validated ncRNA axes rather than for a broad, clinically established ‘autophagy transcriptomic signature.’

### 7.1. The Regulatory Landscape of MicroRNAs

MicroRNAs are short non-coding RNAs that regulate gene expression by binding target transcripts, most commonly within 3′-untranslated regions, leading to transcript degradation and/or translational repression [69,70]. In PitNET research, several miRNAs have been associated with tumor phenotype and treatment resistance, but only a subset has been directly linked to autophagy through validated molecular targets.

The best-characterized example is *miR-93*. In dopamine-agonist-resistant prolactinomas, *miR-93* was increased while its validated target *ATG7* was reduced; experimental overexpression of *miR-93* decreased autophagy and reduced cabergoline cytotoxicity in MMQ and GH3 cells, whereas manipulation of *ATG7* supported the causal relationship [60]. This provides direct evidence that the *miR-93*/*ATG7* axis can contribute to cabergoline resistance. Other miRNAs, including *miR-142-3p*, have been reported as dysregulated in dopamine-agonist-resistant prolactinomas, but a specific causal role in autophagic flux has not been established [72].

### 7.2. Long Non-Coding and Circular RNAs: ceRNA and Exosomal Networks

LncRNAs are transcripts longer than approximately 200 nucleotides that do not encode conventional proteins and can regulate gene expression through diverse mechanisms, including transcriptional regulation, RNA-protein interactions, and competitive endogenous RNA (ceRNA) networks [69,71,73]. In PitNETs, several lncRNAs have been linked to proliferation, apoptosis, signaling pathways, and treatment response, but their effects on autophagy are not uniformly tumor-promoting or tumor-suppressive.

PitNET-specific studies also establish an *H19*-mTORC1/4E-BP1 regulatory axis. *H19* suppresses pituitary tumor growth by binding 4E-BP1, disrupting its interaction with Raptor, and reducing mTORC1-mediated 4E-BP1 phosphorylation [74]. Exosomal *H19* can transfer this growth-inhibitory effect between pituitary tumor cells, while changes in plasma exosomal *H19* have been associated with medical response in prolactinoma [75]. The *H19*/*miR-93a*/*ATG7* axis provides a complementary autophagy-related mechanism: *H19* can increase *ATG7* expression by restraining *miR-93a* activity, thereby enhancing autophagy-related responses and dopamine-agonist sensitivity in pituitary tumor models [76]. The available data support a role in pharmacological sensitivity rather than a broad claim that *H19* normally regulates pituitary physiology or that its expression alone predicts clinical response in individual patients.

*CLRN1-AS1* illustrates the opposite functional direction. In a study of 42 prolactinoma samples and experimental models, *CLRN1-AS1* was downregulated in tumors, suppressed proliferation, promoted apoptosis, and inhibited autophagy [77]. Mechanistically, *CLRN1-AS1* acted as a ceRNA for *miR-217*, increasing *DKK1* and suppressing Wnt/β-catenin signaling; FOXP1 functioned as a transcriptional suppressor of *CLRN1-AS1* [77]. Thus, *CLRN1-AS1* should not be described simply as a generic enhancer of beneficial autophagy.

Earlier work had already implicated *circOMA1* in cabergoline resistance through suppression of ferroptosis via the *miR-145-5p*/*GCLM* axis [78]. A 2026 study extended this framework to exosomal transfer. *circOMA1* was increased in cabergoline-resistant prolactinoma tissue and acted as a *miR-145-5p* sponge, increasing *KBTBD7* and promoting DRD2 ubiquitination and degradation. This attenuated cabergoline-induced autophagy and AKT pathway inhibition; the autophagy phenotype was supported by LC3-II/LC3-I and p62 measurements, ultrastructural analysis, and RFP-GFP-LC3 imaging. Exosomal *circOMA1* also transmitted cabergoline resistance in vitro and in vivo. Plasma exosomal *circOMA1* was evaluated in a broader cohort of 219 patients with PitNETs, providing translational biomarker context, although the resistance mechanism itself remains primarily supported by experimental models [61]. Together, these ncRNA studies emphasize that the biological consequence of autophagy modulation depends on the specific regulatory circuit (Table 3).

**Figure 1:**
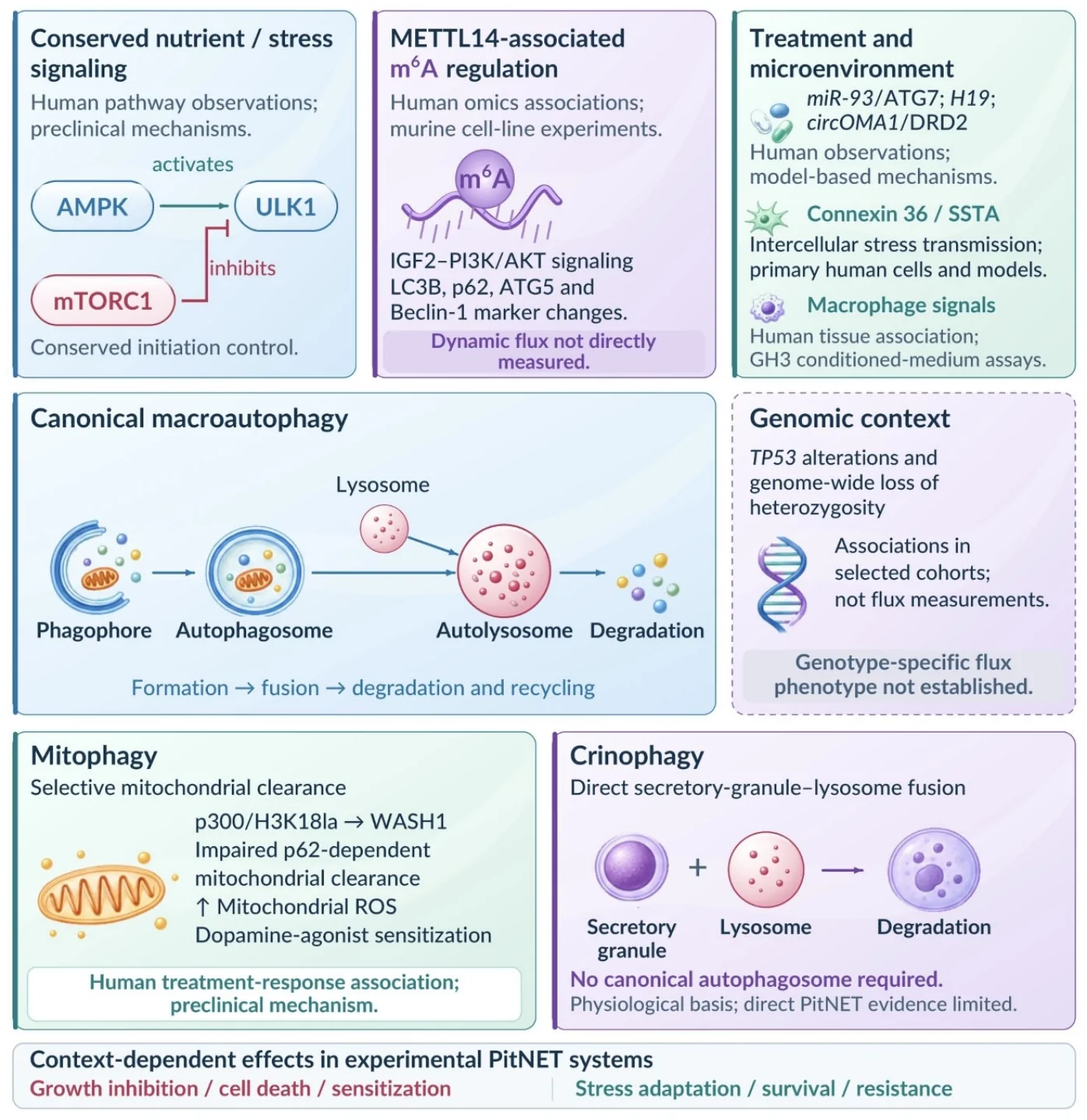
Autophagy in PitNETs: mechanisms and evidence context. Arrowheads indicate activation or process progression; the blunt-ended mTORC1 connector indicates inhibition. The upward arrow denotes increased mitochondrial ROS. Colors distinguish biological topics, not evidence grades. Organelles are schematic and are not drawn to scale. Abbreviations: AKT, protein kinase B; AMPK, AMP-activated protein kinase; ATG5/ATG7, autophagy-related proteins 5/7; DRD2, dopamine receptor D2; GH3, the rat pituitary tumor cell line used in the cited experiments; H3K18la, histone H3 lysine 18 lactylation; IGF2, insulin-like growth factor 2; LC3B, microtubule-associated protein 1 light chain 3 beta; m^6^A, N^6^-methyladenosine; METTL14, methyltransferase 14, an m^6^A writer-complex component; miR, microRNA; mTORC1, mechanistic target of rapamycin complex 1; p300, histone acetyltransferase p300; p62, sequestosome 1; PI3K, phosphoinositide 3-kinase; PitNET, pituitary neuroendocrine tumor; ROS, reactive oxygen species; SSTA, somatostatin analogue; *TP53*, tumor protein p53 gene; ULK1, Unc-51-like autophagy-activating kinase 1; WASH1, WASH complex subunit 1. *H19* and *circOMA1* denote the respective non-coding RNA transcripts.

The mechanisms discussed in Section 3, Section 4, Section 5, Section 6 and Section 7 and their respective evidence contexts are summarized in Figure 1.

Conserved nutrient/stress signaling is distinguished from observations of pathway activity in human PitNETs [32,33,34,35,36,37]. METTL14-associated regulation combines human omics associations and murine cell-line experiments; autophagy-related marker changes do not establish dynamic flux [51]. Treatment and microenvironmental examples include *miR-93*/ATG7 [60], *H19* [74,75,76], *circOMA1*/DRD2 [61], Connexin 36-associated stress transmission during somatostatin-analogue treatment [62], and macrophage-conditioned signals [52]. Canonical macroautophagy proceeds through phagophore expansion, autophagosome–lysosome fusion, and autolysosomal degradation [26,30,31]. *TP53* and genome-wide loss-of-heterozygosity findings are presented separately as associations in selected cohorts, not measurements of autophagic flux [40,41,42,43]. The mitophagy panel depicts the WASH1-related branch of the p300/H3K18la response: impaired p62-dependent mitochondrial clearance is linked to mitochondrial ROS accumulation and dopamine-agonist sensitization in experimental systems, whereas human findings concern treatment–response associations [58]. Crinophagy involves direct secretory-granule–lysosome fusion without a canonical autophagosome; its physiological and experimental basis should not be equated with direct validation in human PitNETs [22,63,66,67]. Outcomes represent different experimental contexts, not a demonstrated reversible switch. Static LC3/p62 abundance and autophagosome counts alone do not establish dynamic flux [30]. In these schematics, italic symbols denote genes or RNA species; protein and complex labels are roman. ATG7 and DRD2 in the RNA-associated panel denote protein effectors. Modification labels, including H3K18la and m^6^A, are roman.

## 8. Autophagy in Standard-Therapy Response and Clinical Translation

Medical management differs substantially among functioning PitNET subtypes. Dopamine agonists, particularly cabergoline, are first-line treatment for most prolactinomas, whereas somatostatin receptor ligands are central medical therapies for acromegaly and TSH-secreting PitNETs in appropriate clinical settings [81,82,83,84,85]. Most prolactinomas respond to dopamine agonists, but primary or secondary resistance occurs in a clinically important subset and is defined using biochemical and tumor-response criteria rather than by a single universal percentage [82,86]. Refractory disease may require surgery, radiotherapy, or additional medical strategies according to tumor subtype and clinical behavior [81,86,87].

### 8.1. The Mechanics of Dopamine Agonists and Somatostatin Analogues

Dopamine agonists act predominantly through dopamine D2 receptors on lactotroph tumor cells, reducing prolactin secretion and usually restraining tumor growth [81,82,86]. Resistance is multifactorial and has been associated with reduced D2-receptor expression or signaling, altered receptor isoforms, cytoskeletal and trafficking abnormalities, and downstream molecular changes [81,86]. These mechanisms are heterogeneous; severe receptor loss should not be presented as the single dominant mechanism in all resistant tumors.

Somatostatin receptor ligands act principally through SSTR2 and SSTR5 in relevant PitNET subtypes. Treatment response is influenced by receptor abundance, membrane localization, receptor trafficking, and downstream signaling [81,85]. Rare receptor variants, including reported *SSTR5* alterations, may influence signaling in selected cases, but receptor mutation or truncation is not regarded as the predominant explanation for somatostatin-ligand resistance across PitNETs [81].

Human tissue data also provide limited treatment-exposure evidence for autophagy-related changes. In a retrospective pilot study comparing 11 patients with acromegaly who received preoperative somatostatin-analogue treatment with 9 untreated patients, treated tumors showed higher TUNEL, caspase-3 and ATG5 immunopositivity, lower Beclin-1 and survivin immunopositivity, and a lower Ki-67 index. Because these observations were based on static tissue markers rather than dynamic-flux measurements, they support treatment-associated remodeling of apoptosis/autophagy-related proteins rather than direct demonstration of increased autophagic flux in patients [88].

ER-stress/autophagy signaling has also been examined in relation to somatostatin-analogue resistance in somatotroph PitNET models. In primary human GH-adenoma cells and GH3/GH4 cell lines, somatostatin-analogue treatment induced endoplasmic-reticulum stress and autophagy-related responses assessed with molecular readouts and tandem mRFP-GFP-LC3 imaging. Endoplasmic-reticulum-stress signals were transmitted between tumor cells and reduced subsequent responsiveness to somatostatin analogues; Connexin 36 mediated this intercellular transfer, while the Connexin 36 blocker quinine enhanced somatostatin-analogue activity in xenograft and syngeneic models. These findings support an ER-stress/Connexin 36/autophagy-associated resistance mechanism but do not establish clinical efficacy of Connexin 36 blockade [62].

### 8.2. Autophagy-Related Cell Death and Autophagic Flux Blockade

Autophagy-related mechanisms have been implicated in dopamine-agonist-induced cell death. In experimental pituitary tumor models, bromocriptine increased autophagic activity and produced cell death that was attenuated by autophagy inhibition, supporting an autophagy-dependent component of its antitumor effect [89]. However, subsequent comparative experiments in GH3 and MMQ cells yielded a different functional interpretation: although bromocriptine induced autophagy-related changes, pharmacological autophagy inhibition with 3-MA did not significantly reverse its growth-inhibitory effect, whereas cabergoline cytotoxicity was more clearly autophagy-dependent; bromocriptine-induced cell death was instead predominantly associated with apoptosis and ERK/EGR1 signaling [90]. The extent to which these experimentally proposed cell-death mechanisms contribute to tumor shrinkage in patients remains uncertain and should be distinguished from established endocrine effects mediated through D2-receptor signaling.

Cabergoline has also been linked to autophagy-dependent cell death in pituitary tumor models. Experimental studies reported suppression of mTOR signaling, increased autophagosome formation, and impaired completion of late autophagic degradation, resulting in accumulation of autophagic structures and cell death [57]. DEPTOR, an endogenous mTOR-interacting protein, can inhibit pituitary tumor cell proliferation and increase sensitivity to dopamine agonists through autophagy-related mechanisms [91]. These data support a mechanistic connection between cabergoline and autophagy but do not imply that late-flux blockade is the only antitumor action of cabergoline.

DRD5 provides an additional experimentally supported link between dopaminergic signaling and autophagy. Activation of DRD5 with SKF83959 increased ROS, inhibited mTOR signaling, induced autophagic structures and late-stage degradative impairment, and suppressed pituitary tumor-cell growth. Knockdown of *ATG5*, *ATG7*, or *BECN1* attenuated the growth-inhibitory effect, and SKF83959 reduced viability in four of five primary human pituitary tumor cultures, with response associated with DRD5 expression [92]. These data provide stronger PitNET-specific evidence than receptor-level extrapolation alone, while remaining insufficient to establish clinical efficacy.

Resistance can emerge through several distinct molecular routes. The *miR-93*/*ATG7* axis can reduce autophagy-related cabergoline cytotoxicity [60]. More recently, cabergoline-induced NDFIP1 upregulation was shown to activate AKT/mTOR signaling and reduce cabergoline sensitivity in experimental PitNET models; NDFIP1 expression also correlated with tumor size in tissue-based analyses [93]. Because that study did not directly quantify dynamic autophagic flux, the NDFIP1 finding is best interpreted as an mTOR-linked resistance mechanism rather than direct evidence of altered flux. In parallel, the 2026 p300/H3K18-lactylation study identifies mitophagy and mitochondrial ROS handling as another modifiable determinant of dopamine-agonist response [58]. A 2026 study also identified exosome-delivered *circOMA1* as a DRD2-centered resistance mechanism: *circOMA1* sponged *miR-145-5p*, increased KBTBD7-mediated DRD2 ubiquitination and degradation, attenuated cabergoline-induced autophagy and AKT suppression, and transmitted resistance through exosomes in experimental models [61]. The study additionally assessed plasma exosomal *circOMA1* in 219 patients with PitNETs, supporting biomarker relevance but not constituting clinical validation of the autophagy resistance mechanism. These findings provide more specific evidence than the untested proposition that resistant PitNETs generally survive by genetically increasing lysosomal clearance.

### 8.3. Chloroquine and Hydroxychloroquine: Late-Stage Autophagy Inhibition

Chloroquine (CQ) and hydroxychloroquine (HCQ) are established antimalarial and immunomodulatory drugs that have been repurposed experimentally as late-stage autophagy inhibitors [94,95,96]. They should not be described as drugs approved specifically for autophagy inhibition in cancer. Their lysosomotropic properties can interfere with lysosomal function and autophagic degradation, which has motivated combination strategies in tumor models.

In pituitary tumor models, combining cabergoline with CQ produced enhanced antitumor effects. Lin and colleagues demonstrated activity in rat pituitary tumor cell lines, primary human pituitary tumor cultures, and in vivo models, with accumulation of autophagy-related proteins and enhanced apoptotic signaling [97]. These findings provide a strong preclinical rationale for late-stage autophagy inhibition in selected dopamine-agonist-resistant settings, while remaining distinct from proof of routine clinical efficacy.

Clinical evidence remains very limited. A 2022 report described a single patient with a cabergoline-resistant lactotroph PitNET who showed a favorable response to the addition of HCQ [98]. This observation is hypothesis-generating and cannot establish efficacy or safety for the broader resistant-prolactinoma population. A clinical study was registered to evaluate cabergoline combined with CQ/HCQ (NCT03400865); the registry currently lists the study status as “Unknown status”, the last update was posted on 16 October 2018, and no tabular study results have been posted [99]. Controlled prospective studies are therefore still required.

Several barriers currently limit translation of CQ/HCQ or other lysosome-directed strategies. These agents have pleiotropic lysosomal and non-autophagic effects, so target engagement cannot be inferred from systemic exposure alone; sustained dosing may also be limited by toxicity, while the intratumoral concentrations required to inhibit lysosomal degradation in human PitNETs are unknown. In addition, no validated biomarker currently identifies patients whose tumors are dependent on a therapeutically targetable autophagy state. Future trials should therefore incorporate pharmacodynamic evidence of lysosomal/autophagic target engagement, rational combination dosing, toxicity monitoring, and pre-specified molecular or flux-based selection criteria rather than treating autophagy inhibition as a uniform class effect [30,94,95,96].

### 8.4. Autophagy and Temozolomide Resistance in Aggressive PitNETs

Temozolomide (TMZ) has a defined role in aggressive pituitary tumors and pituitary carcinomas rather than in PitNETs generally. The 2025 European Society of Endocrinology guideline recommends TMZ monotherapy as first-line chemotherapy after documented tumor progression despite standard multimodal management; contemporary clinical reviews likewise emphasize its role in aggressive or metastatic disease rather than in routine PitNET management [87,100]. Resistance remains a major clinical problem. PitNET-specific autophagy evidence is currently limited to preclinical work: in GH3 cells, HIF-1α knockdown altered presenilin-1-dependent lysosomal acidification, reduced TMZ-induced autophagic progression assessed with tandem fluorescent LC3 and lysosomal readouts, and increased TMZ antitumor activity [101]. This finding provides a mechanistic rationale for studying lysosomal/autophagy state as a modifier of TMZ response, but it has not been validated as a predictive biomarker in human aggressive PitNETs. Longitudinal studies comparing pretreatment and TMZ-resistant human tumors, ideally with dynamic or ex vivo flux-compatible assays, are needed before autophagy-directed TMZ sensitization can be tested clinically.

## 9. Emerging Autophagy-Targeted and Sensitization Strategies

Most pharmacological strategies that directly manipulate autophagy in PitNETs remain preclinical. Their translational value depends not only on whether a compound induces or inhibits autophagy, but also on tumor lineage, baseline pathway activity, the stage of the autophagic process affected, and the interaction with standard endocrine therapy.

### 9.1. ACT001 (Dimethylamino-Micheliolide): A Multi-Targeted Autophagic Inducer

Mechanistic experiments linked ACT001 to MEK4/MAPK signaling with JNK/P38 activation and showed that *ATG7* knockdown or early autophagy inhibition attenuated its cytotoxic effect; dopamine-agonist-resistant models additionally implicated AMPK, EGR1 and mTOR signaling [102,103]. Because the autophagy assessment was dominated by autophagosome formation, LC3/p62 changes and functional interference rather than a comprehensive dynamic-flux design, these studies support autophagy-dependent antitumor activity without defining a universal flux phenotype.

ACT001 therefore illustrates the broader principle developed below: the therapeutic meaning of ‘autophagy induction’ depends on pathway completion, tumor lineage, and the concurrent stress imposed by the drug. The principal therapeutic strategies, the autophagy step they affect, and their translational status are summarized in Table 4.

**Figure 2:**
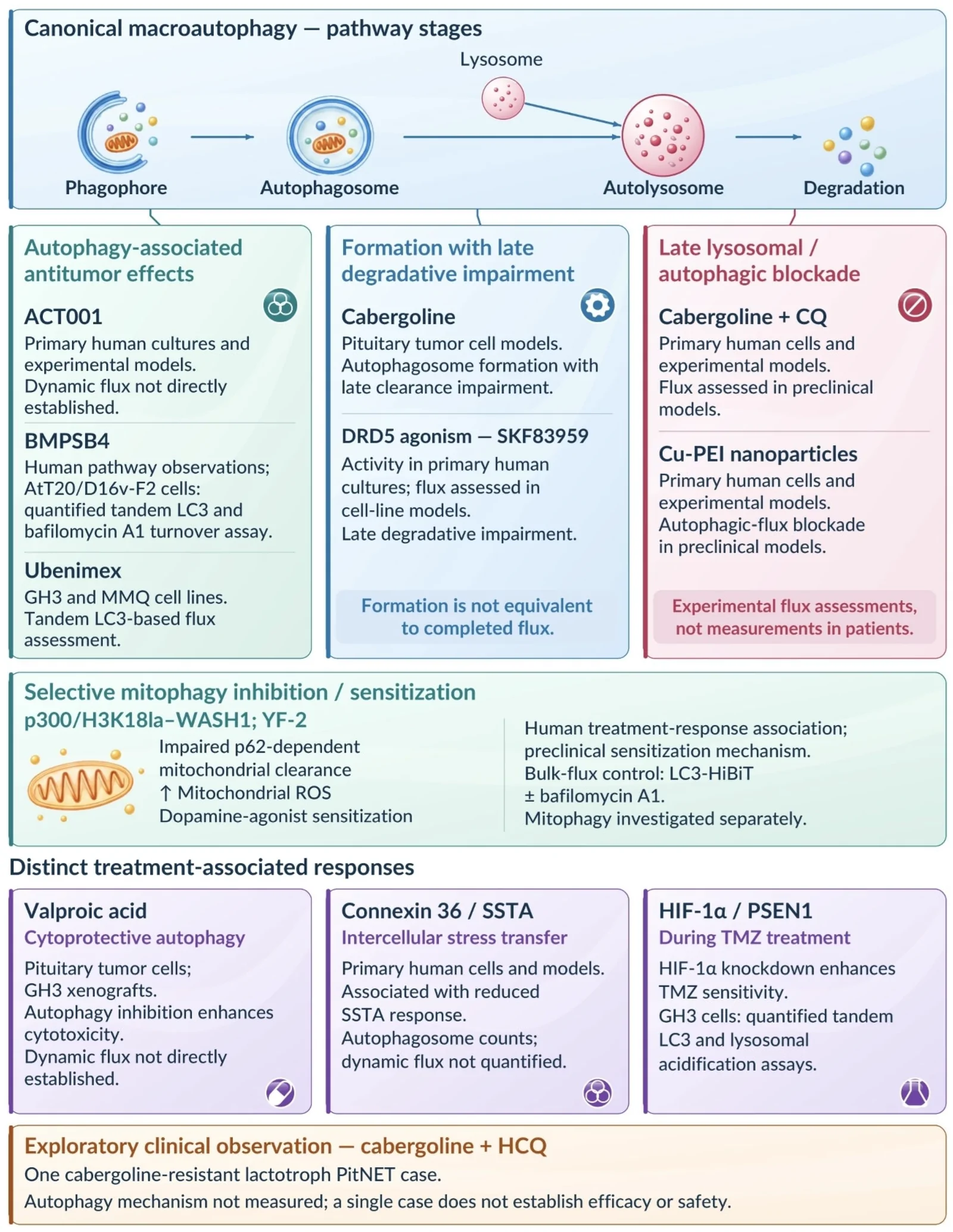
Therapeutic modulation of autophagy in PitNETs. Arrowheads in the upper pathway indicate progression through canonical macroautophagy. Colored panels and connectors organize interventions by the autophagy-related response or experimental context described; they do not indicate a common molecular target, an identical precise site of action, or formal evidence grades. The upward arrow denotes increased mitochondrial ROS, and ± indicates measurements performed with and without bafilomycin A1. Organelles and pictograms are schematic. Abbreviations: ACT001, dimethylamino-micheliolide; BMPSB4, bone morphogenetic protein signaling agonist SB4; CQ, chloroquine; Cu-PEI, copper-polyethyleneimine; DRD5, dopamine receptor D5; H3K18la, histone H3 lysine 18 lactylation; HCQ, hydroxychloroquine; HIF-1α, hypoxia-inducible factor 1 alpha; LC3, microtubule-associated protein 1 light chain 3; LC3-HiBiT, HiBiT-tagged LC3 reporter; p300, histone acetyltransferase p300; p62, sequestosome 1; PitNET, pituitary neuroendocrine tumor; PSEN1, presenilin 1; ROS, reactive oxygen species; SSTA, somatostatin analogue; TMZ, temozolomide; WASH1, WASH complex subunit 1. GH3, MMQ, and AtT20/D16v-F2 denote the cited pituitary cell-line models; SKF83959 is the compound used to investigate DRD5 agonism, and YF-2 is the p300 histone-acetyltransferase-domain activator investigated in the cited study.

Figure 2 summarizes the autophagy-related effects, experimental contexts, and flux assessments of selected interventions.

Representative examples include autophagy-associated antitumor effects with ACT001 [102,103], BMPSB4 [48], and ubenimex [108]; autophagosome formation with late degradative impairment with cabergoline [57] or DRD5 agonism [92]; and late lysosomal/autophagic blockade with cabergoline plus CQ [97] or Cu-PEI nanoparticles [110]. In particular, the late-blockade category does not imply a universal point of inhibition after autophagosome–lysosome fusion. Model descriptions and flux assessments refer to the specified experimental systems, not to measurements in patients. “Not directly established” or “not quantified” indicates limitations of flux assessment, not absence of autophagy. BMPSB4 experiments include quantified tandem LC3 and bafilomycin A1 turnover assessment in AtT20/D16v-F2 cells [48]. In the p300/H3K18la–WASH1 study, the LC3-HiBiT assay with and without bafilomycin A1 is a bulk-flux control, distinguished from the selective-mitophagy experiments [58]. Valproic acid [107], Connexin 36/SSTA [62], and HIF-1α/PSEN1–TMZ [101] are shown as separate treatment-associated contexts. The clinical panel describes one cabergoline-resistant lactotroph PitNET case treated with cabergoline plus HCQ, without measurement of the autophagy mechanism [98]. A single case does not establish general efficacy or safety. Established endocrine indications are not questioned; specifically autophagy-targeted add-on treatment remains investigational. See Table 4 for the full evidence context.

### 9.2. Additional PitNET-Specific Autophagy-Modulating and Sensitization Strategies

Several additional agents illustrate the heterogeneity of autophagy-related responses. Curcumin increased *miR-206*, inhibited AKT/mTOR signaling and altered autophagy-related proteins in GH3 cells; peripheral-blood *miR-206* measurements in treated patients provide human exposure/biomarker context, but the autophagy mechanism itself remains preclinical and dynamic flux was not established [80,111]. In the human NFPA-derived folliculostellate PDFS line, SZ-685C was linked to a *miR-340-3p*/*PPP1CB*/ERK axis and static Beclin-1, LC3, LAMP1 and p62 changes; this non-endocrine-lineage model therefore supports a mechanistic hypothesis rather than a flux-defined clinical strategy [79].

Ubenimex provides stronger methodological evidence for treatment-induced flux: in GH3 and MMQ cells, transmission electron microscopy and tandem mRFP-GFP-LC3 imaging documented autophagosome-to-autolysosome progression, and pharmacological experiments supported a contribution of autophagy to reduced viability alongside ROS/ERK-dependent apoptosis [108]. Celastrol likewise inhibited corticotroph-cell proliferation and xenograft growth with AKT/mTOR-associated apoptosis and autophagy, although flux characterization was more limited [109].

Other PitNET-specific interventions demonstrate that late-stage impairment and autophagy induction are not interchangeable. In an estradiol-induced rat lactotroph model, 15d-PGJ2 reduced pituitary size and prolactin while producing LC3-II/p62 accumulation and reduced LAMP1, consistent with impaired late processing but without direct turnover quantification [104]. In GH-secreting models, SPHK1/S1P/S1PR2 signaling suppressed autophagy and apoptosis; genetic or pharmacological S1PR2 blockade reduced tumor growth and GH secretion, with human data supporting pathway relevance but functional autophagy evidence derived mainly from experimental models [105].

PPARγ-directed and epigenetic approaches further illustrate treatment-specific effects. Rosiglitazone promoted autophagy and apoptosis through 15-PGDH-dependent signaling in GH3 and primary human somatotroph cultures, with qualitative tandem mCherry-GFP-LC3 imaging supporting autophagy-related changes, but without reported quantitative flux analysis; a 19-patient uncontrolled single-arm pilot also showed biochemical drug effects, but the autophagy mechanism was not tested clinically [106]. Conversely, valproic acid induced cytoprotective autophagy in GH3 and xenograft models, and autophagy inhibition enhanced VPA-mediated growth suppression and apoptosis, providing a direct example in which treatment-induced autophagy can protect tumor cells [107].

### 9.3. A Context-Dependent Therapeutic Framework: Why Both Autophagy Activation and Inhibition Can Be Antitumor

The apparently opposing therapeutic results can be reconciled by distinguishing at least four variables. First, autophagy-related responses can be cytoprotective under treatment stress. In the valproic acid experiments, autophagy inhibition increased drug cytotoxicity, although dynamic flux was not directly established [107]. Second, strong autophagy induction can contribute to growth inhibition or cell death when the response proceeds sufficiently or is coupled to other stress pathways, as illustrated by cabergoline and ACT001 models [57,102,103]. Third, inhibition at a late lysosomal step is biologically different from suppressing initiation: cabergoline plus chloroquine and Cu-PEI models exploit impaired degradation or clearance rather than simply producing a lower number of autophagosomes [97,110]. Fourth, selective pathways can behave differently from bulk autophagy; in the p300/H3K18-lactylation study, impaired p62-dependent mitophagy promoted retention of damaged mitochondria, sustained mitochondrial ROS, and enhanced dopamine-agonist-induced apoptosis [58]. Tumor lineage, baseline pathway state, drug dose, timing, and concomitant therapy further modify these effects. The relevant unit of interpretation is therefore the tumor–treatment–flux state, not a binary label of ‘high’ or ‘low’ autophagy.

### 9.4. Translational Priorities and Broader Neuroendocrine Context

Three practical priorities follow from the current evidence. First, lineage-specific autophagy biomarkers should be validated in human PitNET tissue and paired primary/ex vivo models using methods that distinguish formation from degradation. Second, longitudinal sampling of treatment-resistant prolactinomas and somatotroph tumors should link dynamic pathway state to defined drug exposure and clinical response. Third, aggressive PitNETs should be studied prospectively before and after temozolomide with integrated genomic, epigenetic, immune-microenvironmental, and lysosomal/autophagy measurements; recent multi-omic and human immune-microenvironment studies reinforce the importance of lineage and tissue context without themselves establishing autophagic flux [43,53,54]. More broadly, biomarker-driven and pathway-directed trial designs are expanding across neuroendocrine neoplasms [112], but gastroenteropancreatic neuroendocrine tumors are biologically and clinically distinct from PitNETs. Their trial experience should therefore inform study design and endpoint selection rather than be used as mechanistic evidence for pituitary disease.

## 10. Conclusions

The principal contribution of this review is to place PitNET-autophagy findings within an explicit hierarchy of biological context, experimental model and flux methodology. The literature does not support a single model in which autophagy is uniformly tumor-suppressive or tumor-promoting. Instead, the effect depends on lineage, treatment, the selective cargo involved, and whether the pathway is initiated, completed or blocked downstream. This framework also separates PitNET-specific evidence from mechanisms imported from general cancer or endocrine physiology and helps explain why static LC3, p62 or autophagosome measurements can lead to apparently contradictory interpretations [30,31,47,56,57,58].

The priorities outlined in Section 9.4 converge on linking dynamic autophagy measurements to treatment resistance and clinical response in human PitNETs. These efforts should be coupled to genomic and epigenetic context, including *TP53*-associated aggressive corticotroph biology, without assuming that a genomic alteration predicts flux unless this is demonstrated experimentally [40,41,42].

Therapeutic translation remains early. Cabergoline plus CQ has substantial PitNET-specific preclinical support, whereas published cabergoline-plus-HCQ clinical evidence is limited to a single case report [97,98]. Other autophagy- or mitophagy-modulating approaches remain predominantly preclinical, and even interventions with human drug-exposure data have not clinically validated autophagy as the causal mechanism. Accordingly, current evidence does not support routine addition of CQ, HCQ or another autophagy inhibitor to surgical management or standard medical therapy for aggressive PitNETs. Autophagy modulation should remain investigational until biomarker-selected prospective studies demonstrate target engagement, acceptable toxicity and clinically meaningful benefit.

## Figures and Tables

**Table 1:** Interpreting autophagy readouts in PitNET research.

Readout	What It Primarily Indicates	Dynamic Flux?	Key Interpretive Limitation
LC3-I/LC3-II (WB/IHC)	Autophagosome-associated LC3 abundance	No	LC3-II may rise because formation increases or degradation is blocked.
p62/SQSTM1	Cargo-receptor abundance/turnover context	No, alone	Expression is transcriptionally regulated; use complementary assays.
TEM	Autophagosome/autolysosome-like structures and cargo morphology	No	A structural snapshot cannot separate formation from clearance.
Tandem mRFP/EGFP-LC3 or mCherry/GFP-LC3	Autophagosome-to-autolysosome progression	Flux-compatible; yes with quantitative controls	Requires validated reporter behavior, quantification, and appropriate controls.
Lysosomal-inhibitor turnover/time course	Formation versus downstream degradation	Yes	Interpretation depends on dose, timing, and inhibitor specificity.
Lysosomal pH, degradative-capacity or mitophagy reporters	Late-stage function or selective cargo clearance	Yes, if designed dynamically	Combine with pathway-specific and viability controls.

Abbreviations: EGFP, enhanced green fluorescent protein; GFP, green fluorescent protein; IHC, immunohistochemistry; LC3, microtubule-associated protein 1 light chain 3; mRFP, monomeric red fluorescent protein; p62/SQSTM1, sequestosome 1; TEM, transmission electron microscopy; WB, Western blotting. LC3-I and LC3-II denote the non-lipidated and lipidated forms of LC3, respectively; mCherry denotes the red fluorescent reporter protein. Dynamic flux indicates that formation and downstream clearance were distinguished experimentally rather than inferred from a single time-point marker.

**Table 2:** Core stages and molecular components of macroautophagy.

Autophagic Phase	Key Molecular Complexes and Proteins	Core Biochemical Function
Initiation	ULK1 complex; membrane-nucleation factors; LLPS reported in mammalian systems	Integrates nutrient/stress signals and initiates phagophore formation.
Nucleation	PI3KC3 complex (Beclin-1, VPS34, VPS15)	Generates PI3P and recruits membrane-remodeling effectors.
Elongation and LC3 lipidation	ATG12-ATG5-ATG16L1; ATG7; ATG3; LC3-I to LC3-II	Supports membrane expansion and conjugation of LC3 to phosphatidylethanolamine.
Closure/maturation	LC3-II-positive autophagosomal membrane	Completes formation of a sealed autophagosome; static LC3-II abundance alone does not quantify flux.
Degradation	Autophagosome-lysosome fusion; lysosomal hydrolases; v-ATPase	Degrades cargo and releases reusable metabolites into the cytosol.

Abbreviations and symbols: ATG, autophagy-related protein; LC3, microtubule-associated protein 1 light chain 3; LLPS, liquid–liquid phase separation; PI3KC3, class III phosphatidylinositol 3-kinase complex; PI3P, phosphatidylinositol 3-phosphate; ULK1, Unc-51-like autophagy activating kinase 1; v-ATPase, vacuolar-type H+-ATPase; VPS, vacuolar protein sorting.

**Table 3:** Selected non-coding RNAs relevant to autophagy or therapeutic response in PitNET studies and models.

Non-Coding RNA	Type	Primary Target/Interactor	PitNET-Specific Evidence and Functional Consequence
*miR-93*	MicroRNA	*ATG7* mRNA	Increased in dopamine-agonist-resistant prolactinomas; suppresses ATG7-dependent autophagy and contributes to cabergoline resistance in experimental models [60].
*H19*	lncRNA	4E-BP1/Raptor/mTORC1; *miR-93a*/*ATG7*	Suppresses pituitary tumor growth by disrupting the 4E-BP1/Raptor interaction, thereby reducing 4E-BP1 recruitment to mTORC1 and phosphorylation [74]; exosomal *H19* transfers growth-inhibitory effects and provides plasma biomarker/drug-response context [75]; as a ceRNA, increases *ATG7* and enhances dopamine-agonist sensitivity [76].
*CLRN1-AS1*	lncRNA	*miR-217*/*DKK1*/Wnt/β-catenin	Tumor-suppressive in prolactinoma models; reduces proliferation, promotes apoptosis, and inhibits autophagy; transcriptionally suppressed by FOXP1 [77].
*miR-142-3p*	MicroRNA	Autophagy-linked target not established	Reported as dysregulated in dopamine-agonist-resistant prolactinoma; a direct causal role in autophagic flux remains unresolved [72].
*miR-340-3p*	MicroRNA	*PPP1CB*/ERK	Downregulated by SZ-685C in the human NFPA-derived folliculostellate PDFS cell line (non-endocrine-lineage model); *miR-340-3p* overexpression attenuates SZ-685C-associated growth inhibition and autophagy-related marker changes. Static readouts support a preclinical PPP1CB/ERK-autophagy-associated axis, not definitive dynamic flux [79].
*miR-206*	MicroRNA	AKT/mTOR signaling; direct target not established	Curcumin-associated *miR-206* upregulation in GH3 cells accompanies AKT/mTOR inhibition and autophagy-related protein changes; peripheral-blood *miR-206* was measured before/after curcumin treatment in prolactinoma patients, but the autophagy mechanism was demonstrated in GH3 cells [80].
*circOMA1*	Circular RNA	*miR-145-5p*/*GCLM*; *miR-145-5p*/*KBTBD7*/ DRD2	Linked to cabergoline resistance through *miR-145-5p*/*GCLM*-mediated ferroptosis suppression [78]; later shown to promote KBTBD7-dependent DRD2 ubiquitination/degradation, attenuate cabergoline-induced autophagy and AKT inhibition, and transmit resistance via exosomes. Plasma exosomal *circOMA1* was also evaluated in 219 PitNET patients [61].

Abbreviations and symbols: AKT, protein kinase B; ATG7, autophagy-related protein 7; ceRNA, competitive endogenous RNA; DKK1, Dickkopf WNT signaling pathway inhibitor 1; GCLM, glutamate-cysteine ligase modifier subunit; DRD2, dopamine receptor D2; ERK, extracellular signal-regulated kinase; FOXP1, forkhead box P1; KBTBD7, kelch repeat and BTB domain-containing protein 7; lncRNA, long non-coding RNA; miR, microRNA; mRNA, messenger RNA; mTOR, mechanistic target of rapamycin; NFPA, non-functioning pituitary adenoma; PitNET, pituitary neuroendocrine tumor; PPP1CB, protein phosphatase 1 catalytic subunit beta; 4E-BP1, eukaryotic translation initiation factor 4E-binding protein 1; mTORC1, mechanistic target of rapamycin complex 1; Raptor, regulatory-associated protein of mTOR. GH3 denotes the rat pituitary tumor cell line; PDFS denotes the human NFPA-derived folliculostellate cell line used in the cited study.

**Table 4:** Selected PitNET-specific interventions and pathways linked to autophagy: models, flux assessment, and evidence context.

Intervention/Pathway	PitNET-Specific Model or Human Evidence	Autophagy-Related Finding	Main Outcome and Evidence Context	Dynamic Autophagic Flux Assessment	Evidence Type/Human Proximity
Cabergoline + CQ; cabergoline + HCQ case	Rat pituitary cell lines; primary human PitNET cultures; in vivo models; one HCQ-treated resistant lactotroph PitNET case	CQ impairs late lysosomal/autophagic degradation; flux mechanism not demonstrated in the HCQ case	Enhanced cabergoline effect preclinically; HCQ clinical evidence remains one case [94,97,98].	Yes—CQ preclinical; No—HCQ case	Clinical case report + preclinical mechanism
DRD5 agonism (SKF83959)	Pituitary tumor cell lines; human PitNET tissue; 5 primary human cultures	ROS/mTOR inhibition with autophagy induction and late degradative impairment; *ATG5*/*ATG7*/*BECN1* dependence	Growth suppression in 4/5 primary cultures; no clinical efficacy data [92].	Yes	Human tissue/primary cultures + preclinical mechanism
Connexin 36/ER-stress transmission with SSTA	Primary human GH-PitNET cells; GH3/GH4; xenograft and syngeneic models	SSTA-associated ER stress and autophagy; Cx36-mediated intercellular stress transfer reduces subsequent response	Quinine enhanced SSTA activity in vivo; no clinical efficacy established [62].	No—autophagosome counts only; dynamic flux not quantified	Primary human cells + preclinical mechanism
Preoperative somatostatin-analogue exposure	Human acromegaly tissue: 11 treated vs. 9 untreated patients	Higher ATG5, lower Beclin-1, increased apoptosis markers; static tissue readouts	Treatment-associated remodeling of apoptosis/autophagy proteins, not demonstrated flux [88].	No	Human retrospective tissue exposure
Exosomal *circOMA1*/DRD2 resistance	Human PRL-PitNET tissue; MMQ; exosome assays; xenografts; plasma exosomes in 219 PitNET patients	Cabergoline-induced autophagy and AKT suppression attenuated; tandem reporter and TEM support experimental mechanism	KBTBD7-dependent DRD2 degradation and transmissible resistance; human biomarker context [61].	No—flux-compatible tandem reporter; quantitative dynamic flux not demonstrated	Human tissue/biomarker + preclinical mechanism
p300–H3K18la/YF-2	Human prolactinoma specimens; MMQ/AtT20; xenografts; primary-cell experiments	WASH1 impairs p62-dependent mitophagy; bulk flux measured with LC3-HiBiT ± bafilomycin A1	Low p300 associated with poorer DA responsiveness; YF-2 sensitization remains preclinical [58].	Yes—LC3-HiBiT ± BafA1 bulk-flux assay; mitophagy assessed separately	Human tissue/primary + preclinical mechanism
Drp1/mitochondrial dysfunction/AMPK-ULK1	Human pituitary tissue (10 low- vs. 10 high-proliferation); GH3; in vivo model	Mitochondrial ROS/ATP depletion activates AMPK-ULK1; LC3/p62 changes and 3-MA experiments	Drp1 higher in low-proliferation tumors; overexpression reduced growth in models [37].	No	Human tissue association + preclinical mechanism
BMPSB4	Human Cushing-disease pathway characterization; AtT20/D16v-F2; in vivo corticotroph model	BMP4/SMAD-dependent autophagy documented by TEM and tandem mRFP-EGFP-LC3	Reduced tumor growth and ACTH/corticosterone in preclinical models [48].	Yes	Human pathway characterization + preclinical mechanism
15d-PGJ2/PPARγ	Estradiol-induced rat lactotroph PitNET model	LC3-II/p62 accumulation with reduced LAMP1, consistent with impaired late processing	Reduced pituitary size and serum PRL [104].	No	Animal model only
SPHK1/S1P/S1PR2 blockade	GH-PitNET patient data; GH3; in vivo pharmacological model	S1P/S1PR2 signaling suppresses autophagy/apoptosis; JTE-013 blocks S1PR2	Reduced tumor growth and GH; autophagy mechanism mainly experimental [105].	Yes—CQ-based flux assay (GH3); no human flux	Human pathway association + preclinical mechanism
Rosiglitazone/15-PGDH	Human somatotroph tissue/primary cultures; GH3; xenografts; 19-patient uncontrolled pilot	15-PGDH-dependent autophagy/apoptosis; tandem LC3 imaging in cells	IGF-1 decreased in 19/19 and GH in 16/19; autophagy mechanism remains preclinical [106].	Limited—qualitative tandem reporter; quantitative flux not reported	Uncontrolled clinical pilot + human primary/preclinical mechanism
Valproic acid	GH3 and other PitNET cell models; GH3 xenograft	Cytoprotective autophagy; inhibition increases VPA cytotoxicity	Reduced growth/invasion/PRL; treatment-induced autophagy was protective [107].	No	Preclinical only
ACT001	Pituitary tumor cell lines; primary human cultures; rodent models; DA-resistant models	LC3/p62 changes and autophagy dependence via *ATG7*/3-MA; comprehensive flux not established	Antitumor activity in resistant models [102,103].	No	Human primary cultures + preclinical mechanism
Ubenimex/ROS-ERK	GH3 and MMQ cell lines	TEM and tandem mRFP-GFP-LC3 show autophagosome-to-autolysosome progression	Reduced viability with apoptosis and increased flux [108].	Yes	Cell-line preclinical only
SZ-685C/*miR-340-3p*	Human NFPA-derived folliculostellate PDFS cell line	Beclin-1/LC3/LAMP1/p62 changes; *miR-340-3p*/*PPP1CB*/ERK implicated	Reduced viability, migration, and proliferation; static autophagy readouts [79].	No	Human-derived cell-line preclinical only
Curcumin	GH3 cells; peripheral-blood *miR-206* before/after curcumin in prolactinoma patients	*miR-206* upregulation and AKT/mTOR suppression with static autophagy-related changes	Human drug-exposure/biomarker context; autophagy mechanism demonstrated in GH3 [80].	No	Human exposure/biomarker + preclinical mechanism
Celastrol	AtT20 corticotroph cells; mouse xenografts	Autophagy/apoptosis associated with AKT/mTOR inhibition; flux incompletely characterized	Reduced proliferation/migration and xenograft growth [109].	No	Preclinical only
SOX6-LAMP5-NF-κB axis	Pituitary adenoma tissue; GH3; nude-mouse xenograft	LAMP5 promotes NF-κB-dependent autophagy; predominantly static/autophagosome readouts	*LAMP5* knockdown or *SOX6* overexpression reduced growth [50].	No	Human tissue association + preclinical mechanism
METTL14/m^6^A-*IGF2*/PI3K-AKT	Human bulk/scRNA-seq datasets; TtT/GF and AtT20 murine lines	LC3B-II/I, p62, ATG5, and Beclin-1 changes with PI3K/AKT activation; no direct flux assay	Human invasive-PitNET association with murine mechanistic validation [51].	No	Human omics association + preclinical mechanism
Macrophage-conditioned factors/CTSZ-associated TME	Human somatotroph PitNET tissue; GH3 exposed to macrophage-conditioned medium	Reduced autolysosomes/LC3-II; TGF-β implicated; CTSZ alone did not reproduce phenotype	Human tissue supports CTSZ as a marker; causal autophagy effects are model-based [52].	No	Human tissue association + preclinical mechanism
Cu-PEI nanoparticles	PitNET cell lines; primary PitNET cells; in vivo models	Mitochondrial dysfunction with KLF2-associated autophagic-flux blockade	Copper-dependent tumor suppression; nanomedicine evidence remains preclinical [110].	Yes	Human primary cells + preclinical mechanism
HIF-1α/PSEN1 modulation with TMZ	GH3 cells	HIF-1α knockdown impairs lysosomal acidification and TMZ-induced autophagy via PSEN1	Increased TMZ efficacy; mechanistic evidence only [101].	Yes	Cell-line preclinical only

Abbreviations and symbols: 3-MA, 3-methyladenine; 15d-PGJ2, 15-deoxy-Δ12,14-prostaglandin J2; 15-PGDH, 15-hydroxyprostaglandin dehydrogenase; ACT001, dimethylamino-micheliolide; ACTH, adrenocorticotropic hormone; AKT, protein kinase B; AMPK, AMP-activated protein kinase; ATG5/ATG7, autophagy-related proteins 5/7; ATP, adenosine triphosphate; BafA1, bafilomycin A1; BECN1, Beclin 1; BMP4, bone morphogenetic protein 4; BMPSB4, bone morphogenetic protein signaling agonist SB4; CQ, chloroquine; CTSZ, cathepsin Z; Cu-PEI, copper-polyethyleneimine; Cx36, connexin 36; DA, dopamine agonist; DRD2/DRD5, dopamine receptors D2/D5; Drp1, dynamin-related protein 1; ER, endoplasmic reticulum; ERK, extracellular signal-regulated kinase; GH, growth hormone; H3K18la, histone H3 lysine 18 lactylation; HCQ, hydroxychloroquine; HIF-1α, hypoxia-inducible factor 1 alpha; IGF-1/IGF2, insulin-like growth factor 1/2; KBTBD7, kelch repeat and BTB domain-containing protein 7; KLF2, Krüppel-like factor 2; LAMP1, lysosome-associated membrane protein 1; LC3, microtubule-associated protein 1 light chain 3; LC3B, microtubule-associated protein 1 light chain 3 beta; LC3-HiBiT, HiBiT-tagged LC3 reporter; m^6^A, N^6^-methyladenosine; miR, microRNA; mTOR, mechanistic target of rapamycin; NF-κB, nuclear factor kappa B; NFPA, non-functioning pituitary adenoma; PI3K, phosphoinositide 3-kinase; p300, histone acetyltransferase p300; p62, sequestosome 1; PitNET, pituitary neuroendocrine tumor; PPARγ, peroxisome proliferator-activated receptor gamma; PPP1CB, protein phosphatase 1 catalytic subunit beta; PRL, prolactin; PRL-PitNET, prolactin-secreting pituitary neuroendocrine tumor; PSEN1, presenilin 1; ROS, reactive oxygen species; S1P, sphingosine-1-phosphate; S1PR2, sphingosine-1-phosphate receptor 2; scRNA-seq, single-cell RNA sequencing; SPHK1, sphingosine kinase 1; SSTA, somatostatin analogue; TEM, transmission electron microscopy; TGF-β, transforming growth factor beta; TME, tumor microenvironment; TMZ, temozolomide; ULK1, Unc-51-like autophagy activating kinase 1; VPA, valproic acid; WASH1, WASH complex subunit 1; GFP, green fluorescent protein; LAMP5, lysosome-associated membrane protein 5; METTL14, methyltransferase 14, N^6^-adenosine-methyltransferase non-catalytic subunit; SOX6, SRY-box transcription factor 6; EGFP, enhanced green fluorescent protein; mRFP, monomeric red fluorescent protein; SMAD, SMAD family of signaling proteins. GH3, GH4, MMQ, AtT20, AtT20/D16v-F2, TtT/GF, and PDFS denote the cited pituitary cell-line models; SKF83959, YF-2, JTE-013, and SZ-685C are the compound identifiers used in the cited studies. The final column reports evidence type and proximity to human observation rather than a formal certainty-of-evidence tier. Clinical case reports, uncontrolled clinical pilots, human tissue/omics associations, primary-cell studies, and preclinical mechanisms are therefore identified explicitly; a human or clinical observation does not imply that the autophagy mechanism was demonstrated in patients.

## Data Availability

No new data were created or analyzed in this study.

## References

[1] Asa S.L., Mete O., Perry A., Osamura R.Y. (2022). Overview of the 2022 WHO Classification of Pituitary Tumors. Endocr. Pathol..

[2] Casar-Borota O., Burman P., Lopes M.B. (2025). The 2022 WHO Classification of Tumors of the Pituitary Gland: An Update on Aggressive and Metastatic Pituitary Neuroendocrine Tumors. Brain Pathol..

[3] Tataranu L.G. (2025). A Brief Overview of Molecular Biology in Pituitary Adenomas with a Focus on Aggressive Lesions. Int. J. Mol. Sci..

[4] Ezzat S., Asa S.L., Couldwell W.T., Barr C.E., Dodge W.E., Vance M.L., McCutcheon I.E. (2004). The Prevalence of Pituitary Adenomas: A Systematic Review. Cancer.

[5] Daly A.F., Rixhon M., Adam C., Dempegioti A., Tichomirowa M.A., Beckers A. (2006). High Prevalence of Pituitary Adenomas: A Cross-Sectional Study in the Province of Liège, Belgium. J. Clin. Endocrinol. Metab..

[6] Agustsson T.T., Baldvinsdottir T., Jonasson J.G., Olafsdottir E., Steinthorsdottir V., Sigurdsson G., Thorsson A.V., Carroll P.V., Korbonits M., Benediktsson R. (2015). The Epidemiology of Pituitary Adenomas in Iceland, 1955–2012: A Nationwide Population-Based Study. Eur. J. Endocrinol..

[7] Graffeo C.S., Yagnik K.J., Carlstrom L.P., Lakomkin N., Bancos I., Davidge-Pitts C., Erickson D., Choby G., Pollock B.E., Chamberlain A.M. (2022). Pituitary Adenoma Incidence, Management Trends, and Long-term Outcomes: A 30-Year Population-Based Analysis. Mayo Clin. Proc..

[8] Yang Q., Li X. (2019). Molecular Network Basis of Invasive Pituitary Adenoma: A Review. Front. Endocrinol..

[9] Fu X., Li H., Yu T., Pei Y., Chen X., Li J., Liu K., Liu Y. (2025). The Dual Roles of Autophagy in Pituitary Adenomas: Current Research Status. World Neurosurg..

[10] Liu S., Yao S., Yang H., Liu S., Wang Y. (2023). Autophagy: Regulator of Cell Death. Cell Death Dis..

[11] Mizushima N., Komatsu M. (2011). Autophagy: Renovation of Cells and Tissues. Cell.

[12] Niu X., You Q., Hou K., Tian Y., Wei P., Zhu Y., Gao B., Ashrafizadeh M., Aref A.R., Kalbasi A. (2025). Autophagy in Cancer Development, Immune Evasion, and Drug Resistance. Drug Resist. Updates.

[13] Singh S.S., Vats S., Chia A.Y.Q., Tan T.Z., Deng S., Ong M.S., Arfuso F., Yap C.T., Goh B.C., Sethi G. (2018). Dual Role of Autophagy in Hallmarks of Cancer. Oncogene.

[14] Li X., He S., Ma B. (2020). Autophagy and Autophagy-Related Proteins in Cancer. Mol. Cancer.

[15] Verma A.K., Bharti P.S., Rafat S., Bhatt D., Goyal Y., Pandey K.K., Ranjan S., Almatroodi S.A., Alsahli M.A., Rahmani A.H. (2021). Autophagy Paradox of Cancer: Role, Regulation, and Duality. Oxidative Med. Cell. Longev..

[16] Pizzimenti C., Fiorentino V., Ruggeri C., Franchina M., Ercoli A., Tuccari G., Ieni A. (2024). Autophagy Involvement in Non-Neoplastic and Neoplastic Endometrial Pathology: The State of the Art with a Focus on Carcinoma. Int. J. Mol. Sci..

[17] Pizzimenti C., Fiorentino V., Franchina M., Martini M., Giuffrè G., Lentini M., Silvestris N., Di Pietro M., Fadda G., Tuccari G. (2023). Autophagic-Related Proteins in Brain Gliomas: Role, Mechanisms, and Targeting Agents. Cancers.

[18] Yin E., Satou M., Tateno T. (2025). Targeting Autophagy for Pituitary Tumors. Cancers.

[19] Hu B., Li H., Zhang X. (2021). A Balanced Act: The Effects of GH-GHR-IGF1 Axis on Mitochondrial Function. Front. Cell Dev. Biol..

[20] Csizmadia T., Lőrincz P., Hegedűs K., Széplaki S., Lőw P., Juhász G. (2018). Molecular Mechanisms of Developmentally Programmed Crinophagy in Drosophila. J. Cell Biol..

[21] Picca A., Faitg J., Auwerx J., Ferrucci L., D’Amico D. (2023). Mitophagy in Human Health, Ageing and Disease. Nat. Metab..

[22] Smith R.E., Farquhar M.G. (1966). Lysosome Function in the Regulation of the Secretory Process in Cells of the Anterior Pituitary Gland. J. Cell Biol..

[23] Wei Z., Hu X., Wu Y., Zhou L., Zhao M., Lin Q. (2024). Molecular Mechanisms Underlying Initiation and Activation of Autophagy. Biomolecules.

[24] Yamamoto H., Matsui T. (2024). Molecular Mechanisms of Macroautophagy, Microautophagy, and Chaperone-Mediated Autophagy. J. Nippon Med. Sch..

[25] Griffey C.J., Yamamoto A. (2022). Macroautophagy in CNS Health and Disease. Nat. Rev. Neurosci..

[26] Patergnani S., Missiroli S., Morciano G., Perrone M., Mantovani C.M., Anania G., Fiorica F., Pinton P., Giorgi C. (2021). Understanding the Role of Autophagy in Cancer Formation and Progression Is a Real Opportunity to Treat and Cure Human Cancers. Cancers.

[27] Gómez-Virgilio L., Silva-Lucero M.D.C., Flores-Morelos D.S., Gallardo-Nieto J., Lopez-Toledo G., Abarca-Fernandez A.M., Zacapala-Gómez A.E., Luna-Muñoz J., Montiel-Sosa F., Soto-Rojas L.O. (2022). Autophagy: A Key Regulator of Homeostasis and Disease: An Overview of Molecular Mechanisms and Modulators. Cells.

[28] Pizzimenti C., Curcio A., Fiorentino V., Germanò A., Martini M., Ieni A., Tuccari G. (2025). Immunoexpression of Autophagy-Related Proteins in a Single-Center Series of Sporadic Adult Conventional Clival Chordomas. Oncol. Lett..

[29] Ieni A., Pizzimenti C., Fiorentino V., Franchina M., Germanò A., Raffa G., Martini M., Fadda G., Tuccari G. (2024). Immunohistochemical Profile of p62/SQSTM1/Sequestosome-1 in Human Low- and High-Grade Intracranial Meningiomas. Anal. Cell. Pathol..

[30] Klionsky D.J., Petroni G., Amaravadi R.K., Baehrecke E.H., Ballabio A., Boya P., Bravo-San Pedro J.M., Cadwell K., Cecconi F., Choi A.M.K. (2021). Guidelines for the Use and Interpretation of Assays for Monitoring Autophagy (4th Edition). Autophagy.

[31] Cecenarro L.A., Moyano Crespo G.D., Guido C.B., Pérez P.A., Faure E.E., Picech F., Rossetto S.V., De Battista J.C., Gutiérrez S., Torres A.I. (2023). Ultrastructural and Molecular Evidence of Macroautophagy in Functioning PitNETs and Experimental Pituitary Tumors. Neuroendocrinology.

[32] Monsalves E., Juraschka K., Tateno T., Agnihotri S., Asa S.L., Ezzat S., Zadeh G. (2014). The PI3K/AKT/mTOR Pathway in the Pathophysiology and Treatment of Pituitary Adenomas. Endocr.-Relat. Cancer.

[33] Derwich A., Sykutera M., Bromińska B., Rubiś B., Ruchała M., Sawicka-Gutaj N. (2023). The Role of Activation of PI3K/AKT/mTOR and RAF/MEK/ERK Pathways in Aggressive Pituitary Adenomas—New Potential Therapeutic Approach—A Systematic Review. Int. J. Mol. Sci..

[34] Zhang J., Li X., Zhang Y. (2017). Correlation of NEDD4-1 and PTEN Expression with the Invasive Capacity of Pituitary Adenomas. Mol. Clin. Oncol..

[35] Sinha R.A., Singh B.K., Yen P.M. (2017). Reciprocal Crosstalk Between Autophagic and Endocrine Signaling in Metabolic Homeostasis. Endocr. Rev..

[36] Bizzi M.F., Drummond J.B., Pinheiro S.V.B., Paulino E., Araújo S.A., Soares B.S., Giannetti A.V., Schweizer J.R.O.L., Barry S., Korbonits M. (2024). Activated AMP-Protein Kinase (pAMPK) Is Overexpressed in Human Somatotroph Pituitary Adenomas. Mol. Cell. Endocrinol..

[37] Fan K., Ding X., Zang Z., Zhang Y., Tang X., Pei X., Chen Q., Yin H., Zheng X., Chen Y. (2022). Drp1-Mediated Mitochondrial Metabolic Dysfunction Inhibits the Tumor Growth of Pituitary Adenomas. Oxidative Med. Cell. Longev..

[38] Tasdemir E., Maiuri M.C., Galluzzi L., Vitale I., Djavaheri-Mergny M., D’Amelio M., Criollo A., Morselli E., Zhu C., Harper F. (2008). Regulation of Autophagy by Cytoplasmic p53. Nat. Cell Biol..

[39] Crighton D., Wilkinson S., O’Prey J., Syed N., Smith P., Harrison P.R., Gasco M., Garrone O., Crook T., Ryan K.M. (2006). DRAM, a p53-Induced Modulator of Autophagy, Is Critical for Apoptosis. Cell.

[40] Uzilov A.V., Taik P., Cheesman K.C., Javanmard P., Ying K., Roehnelt A., Wang H., Fink M.Y., Lau C.Y., Moe A.S. (2021). USP8 and TP53 Drivers Are Associated with CNV in a Corticotroph Adenoma Cohort Enriched for Aggressive Tumors. J. Clin. Endocrinol. Metab..

[41] Perez-Rivas L.G., Simon J., Albani A., Tang S., Roeber S., Assié G., Deutschbein T., Fassnacht M., Gadelha M.R., Hermus A.R. (2022). TP53 Mutations in Functional Corticotroph Tumors Are Linked to Invasion and Worse Clinical Outcome. Acta Neuropathol. Commun..

[42] Lin A.L., Rudneva V.A., Richards A.L., Zhang Y., Woo H.J., Cohen M., Tisnado J., Majd N., Wardlaw S.L., Page-Wilson G. (2024). Genome-Wide Loss of Heterozygosity Predicts Aggressive, Treatment-Refractory Behavior in Pituitary Neuroendocrine Tumors. Acta Neuropathol..

[43] Benanteur N., Villa C., Bioletto F., Birtolo M.F., De Murat D., Masliah-Planchon J., Gravrand V., Lebib I., Benkhellat M., Boezennec R. (2026). Genomic Characterization of Aggressiveness in Pituitary Neuroendocrine Tumors. Neuro-Oncology.

[44] Song Z.J., Reitman Z.J., Ma Z.Y., Chen J.H., Zhang Q.L., Shou X.F., Huang C.X., Wang Y.F., Li S.Q., Mao Y. (2016). The Genome-Wide Mutational Landscape of Pituitary Adenomas. Cell Res..

[45] Chen J., Jian X., Deng S., Ma Z., Shou X., Shen Y., Zhang Q., Song Z., Li Z., Peng H. (2018). Identification of Recurrent USP48 and BRAF Mutations in Cushing’s Disease. Nat. Commun..

[46] Cazabat L., Bouligand J., Salenave S., Bernier M., Gaillard S., Parker F., Young J., Guiochon-Mantel A., Chanson P. (2012). Germline AIP Mutations in Apparently Sporadic Pituitary Adenomas: Prevalence in a Prospective Single-Center Cohort of 443 Patients. J. Clin. Endocrinol. Metab..

[47] Qiu C., Yao Y., Hu S., Xu Y. (2024). HES1 Induces ITPR1-Mediated Autophagy to Exert Anti-Metastatic Effects in Pituitary Adenomas. Transl. Cancer Res..

[48] Xu L., Ning R., Du X., Zhang Y., Gu C., Wang B., Bian L., Sun Q., Sun Y., Ren J. (2024). Bone Morphogenetic Protein Signaling Agonist SB4 (BMPSB4) Inhibits Corticotroph Pituitary Neuroendocrine Tumors by Activation of Autophagy via a BMP4/SMADs-Dependent Pathway. ACS Pharmacol. Transl. Sci..

[49] Pattingre S., Tassa A., Qu X., Garuti R., Xiao H.L., Mizushima N., Packer M., Schneider M.D., Levine B. (2005). Bcl-2 Antiapoptotic Proteins Inhibit Beclin 1-Dependent Autophagy. Cell.

[50] Liu Z., Liao X., Zhao H., Ruan B., Jia F., Long R. (2026). The Transcription Factor SOX6 Regulates Autophagy Levels Through LAMP5 to Inhibit the Development of Pituitary Adenomas. Appl. Biochem. Biotechnol..

[51] Yang S., Pu C., Deng C., Bai X., Tian C., Zhang W., Deng K., Duan L., Lu L., Zhu H. (2026). Single-Cell and Machine Learning Approaches Reveal METTL14-Mediated Autophagy via PI3K/AKT Signaling in Invasive PitNET. Cancer Cell Int..

[52] Chen M., Duan L., Miao H., Yang S., Wang L., Gong F., Yao Y., Zhu H. (2026). Macrophage-Secreted Factors Promote Invasiveness in Somatotroph PitNETs: Cathepsin Z as a Macrophage-Associated Marker. Mol. Cell. Endocrinol..

[53] Lamb L.S., Sim H.W., Ramus S.J., Earls P.E., Low J.P., Thompson T., Lenders N.F., McCormack A.I. (2026). Aggressive Pituitary Neuroendocrine Tumors Display a Distinct Immune Microenvironment. J. Clin. Endocrinol. Metab..

[54] Loughrey P.B., McArdle A., Pulsawatdi A.V., McCombe K.D., Greene C., James H., Healy E.G., Corr F., Ross E., Cooke S. (2026). STING Expression in Pituitary Neuroendocrine Tumour Immune Microenvironments. Endocr.-Relat. Cancer.

[55] Xie T., Xie Q., Hu Q., Hu J., Gao Y., Luo R., Yan C., Chen P., Yang Z., Liu Y. (2026). Region-Resolved Integrative Multi-Omic Characterization Reveals Diverse Tumor and Microenvironment Features of Pituitary Neuroendocrine Tumors. Mol. Cell. Proteom..

[56] Satou M., Wang J., Nakano-Tateno T., Teramachi M., Aoki S., Sugimoto H., Chik C., Tateno T. (2024). Autophagy Inhibition Suppresses Hormone Production and Cell Growth in Pituitary Tumor Cells: A Potential Approach to Pituitary Tumors. Mol. Cell. Endocrinol..

[57] Lin S.J., Leng Z.G., Guo Y.H., Cai L., Cai Y., Li N., Shang H.B., Le W.D., Zhao W.G., Wu Z.B. (2015). Suppression of mTOR Pathway and Induction of Autophagy-Dependent Cell Death by Cabergoline. Oncotarget.

[58] Li S., Jiang Q., Wang Q., Li X., Wang Z., Xu L., Luo S., Wang Y., Zhang H., Shu K. (2026). p300-Mediated Histone H3K18 Lactylation Promotes Mitochondrial ROS Accumulation via Mitophagy Inhibition to Potentiate Dopamine Agonists Efficacy in Prolactinomas. Redox Biol..

[59] Buccarelli M., Castellani G., Fiorentino V., Pizzimenti C., Beninati S., Ricci-Vitiani L., Scattoni M.L., Mischiati C., Facchiano F., Tabolacci C. (2024). Biological Implications and Functional Significance of Transglutaminase Type 2 in Nervous System Tumors. Cells.

[60] Wu Z., Cai L., Lu J., Wang C.D., Guan J., Chen X., Wu J., Zheng W., Wu Z., Li Q. (2019). MicroRNA-93 Mediates Cabergoline Resistance by Targeting ATG7 in Prolactinoma. J. Endocrinol..

[61] Rao Q., Zhu D., Wang Z., Zhang S., Li X., Tagilapalli S., Wang N., Hu W., Chen W., Zhu Y. (2026). circOMA1 Delivered by Exosomes Regulates DRD2-Mediated Prolactinoma Resistance. Int. J. Mol. Med..

[62] Wang Z., Lei Z., Wang Q., Jiang Q., Zhang Z., Liu X., Xing B., Li S., Guo X., Liu Y. (2024). Connexin 36 Mediated Intercellular Endoplasmic Reticulum Stress Transmission Induces SSTA Resistance in Growth Hormone Pituitary Adenoma. Int. J. Biol. Sci..

[63] Szenci G., Csizmadia T., Juhász G. (2023). The Role of Crinophagy in Quality Control of the Regulated Secretory Pathway. J. Cell Sci..

[64] Poole L.P., Macleod K.F. (2021). Mitophagy in Tumorigenesis and Metastasis. Cell. Mol. Life Sci..

[65] Zhang Y., Ma X., Liu C., Bie Z., Liu G., Liu P., Yang Z. (2024). Identification of HSPD1 as a Novel Invasive Biomarker Associated with Mitophagy in Pituitary Adenomas. Transl. Oncol..

[66] Szenci G., Glatz G., Takáts S., Juhász G. (2024). The Ykt6-Snap29-Syx13 SNARE Complex Promotes Crinophagy via Secretory Granule Fusion with Lamp1 Carrier Vesicles. Sci. Rep..

[67] Li M., Feng F., Feng H., Hu P., Xue Y., Xu T., Song E. (2022). VAMP4 Regulates Insulin Levels by Targeting Secretory Granules to Lysosomes. J. Cell Biol..

[68] Garcia E.A., Trivellin G., Aflorei E.D., Powell M., Grieve J., Alusi G., Pobereskin L., Shariati B., Cudlip S., Roncaroli F. (2013). Characterization of SNARE Proteins in Human Pituitary Adenomas: Targeted Secretion Inhibitors as a New Strategy for the Treatment of Acromegaly?. J. Clin. Endocrinol. Metab..

[69] Yan H., Bu P. (2021). Non-Coding RNA in Cancer. Essays Biochem..

[70] Yao H., Han B., Zhang Y., Shen L., Huang R. (2019). Non-Coding RNAs and Autophagy. Adv. Exp. Med. Biol..

[71] Wang L., Xu D. (2023). Regulation of Long Noncoding RNAs in the Pathogenesis and Clinical Implications of Pituitary Adenomas. Immun. Inflamm. Dis..

[72] Wan X., Yan Z., Tan Z., Cai Z., Qi Y., Lu L., Xu Y., Chen J., Lei T. (2022). MicroRNAs in Dopamine Agonist-Resistant Prolactinoma. Neuroendocrinology.

[73] Pecci V., Troisi F., Aiello A., De Martino S., Carlino A., Fiorentino V., Ripoli C., Rotili D., Pierconti F., Martini M. (2024). Targeting of H19/Cell Adhesion Molecules Circuitry by GSK-J4 Epidrug Inhibits Metastatic Progression in Prostate Cancer. Cancer Cell Int..

[74] Wu Z.R., Yan L., Liu Y.T., Cao L., Guo Y.H., Zhang Y., Yao H., Cai L., Shang H.B., Rui W.W. (2018). Inhibition of mTORC1 by lncRNA H19 via disrupting 4E-BP1/Raptor interaction in pituitary tumours. Nat. Commun..

[75] Zhang Y., Liu Y.T., Tang H., Xie W.Q., Yao H., Gu W.T., Zheng Y.Z., Shang H.B., Wang Y., Wei Y.X. (2019). Exosome-Transmitted lncRNA H19 Inhibits the Growth of Pituitary Adenoma. J. Clin. Endocrinol. Metab..

[76] Wu Z., Zheng Y., Xie W., Li Q., Zhang Y., Ren B., Cai L., Cheng Y., Tang H., Su Z. (2020). The Long Noncoding RNA-H19/miRNA-93a/ATG7 Axis Regulates the Sensitivity of Pituitary Adenomas to Dopamine Agonists. Mol. Cell. Endocrinol..

[77] Wang C., Tan C., Wen Y., Zhang D., Li G., Chang L., Su J., Wang X. (2019). FOXP1-Induced lncRNA CLRN1–AS1 Acts as a Tumor Suppressor in Pituitary Prolactinoma by Repressing Autophagy via Inactivation of the Wnt/beta-Catenin Signaling Pathway. Cell Death Dis..

[78] Wu N., Zhu D., Li J., Li X., Zhu Z., Rao Q., Hu B., Wang H., Zhu Y. (2023). CircOMA1 Modulates Cabergoline Resistance by Downregulating Ferroptosis in Prolactinoma. J. Endocrinol. Investig..

[79] Wang X., Wang Z.Y., Chen H.T., Luo Y.Y., Li S.Y., Luo X.M., Yang J.H., Ma Y.X., Jin X.B., Liu J. (2024). SZ-685C Inhibits the Growth of Non-Functioning Pituitary Adenoma by Down-Regulating miR-340-3p and Inducing Autophagy. Heliyon.

[80] Duan J.F., Zhang Q.J., Zhu J., Lu J.H. (2024). Curcumin Affects Autophagy of Prolactinoma Cells by Upregulating miR-206 to Exert Antitumor Effects. J. Biochem. Mol. Toxicol..

[81] Peverelli E., Treppiedi D., Giardino E., Vitali E., Lania A.G., Mantovani G. (2015). Dopamine and Somatostatin Analogues Resistance of Pituitary Tumors: Focus on Cytoskeleton Involvement. Front. Endocrinol..

[82] Inder W.J., Jang C. (2022). Treatment of Prolactinoma. Medicina.

[83] Briet C., Suteau V., Illouz F., Rodien P. (2023). Thyrotropin-Secreting Tumor “TSH-PitNET”: From Diagnosis to Treatment. Ann. d’Endocrinologie.

[84] Shimon I. (2024). Real-World Value of Cabergoline in the Treatment of Acromegaly. Best Pract. Res. Clin. Endocrinol. Metab..

[85] Nagamine T., Fukuda I. (2023). Medical Therapies in Functioning Pituitary Neuroendocrine Tumors (PitNETs). No Shinkei Geka.

[86] Pivonello C., Patalano R., Negri M., Pirchio R., Colao A., Pivonello R., Auriemma R.S. (2022). Resistance to Dopamine Agonists in Pituitary Tumors: Molecular Mechanisms. Front. Endocrinol..

[87] Raverot G., Burman P., Abreu A.P., Heaney A.P., van Hulsteijn L., Lin A.L., Marcus H., McCormack A., Minniti G., Petersenn S. (2025). Revised European Society of Endocrinology Clinical Practice Guideline for the Management of Aggressive Pituitary Tumours and Pituitary Carcinomas. Eur. J. Endocrinol..

[88] Dagistanli F.K., Ozkaya H.M., Kucukyoruk B., Biceroglu H., Metin D., Gazioglu N., Oz B., Kadioglu P., Ozturk M. (2018). Preoperative Somatostatin Analogue Treatment Might Trigger Apoptosis and Autophagy in Tumor Tissues of Patients with Acromegaly: A Pilot Study. Exp. Clin. Endocrinol. Diabetes.

[89] Geng X., Ma L., Li Z., Li Z., Li J., Li M., Wang Q., Chen Z., Sun Q. (2017). Bromocriptine Induces Autophagy-Dependent Cell Death in Pituitary Adenomas. World Neurosurg..

[90] Tang C., Sun R., Wen G., Zhong C., Yang J., Zhu J., Cong Z., Luo X., Ma C. (2019). Bromocriptine and Cabergoline Induce Cell Death in Prolactinoma Cells via the ERK/EGR1 and AKT/mTOR Pathway Respectively. Cell Death Dis..

[91] Yao H., Tang H., Zhang Y., Zhang Q.F., Liu X.Y., Liu Y.T., Gu W.T., Zheng Y.Z., Shang H.B., Wang Y. (2019). DEPTOR Inhibits Cell Proliferation and Confers Sensitivity to Dopamine Agonist in Pituitary Adenoma. Cancer Lett..

[92] Leng Z.G., Lin S.J., Wu Z.R., Guo Y.H., Cai L., Shang H.B., Tang H., Xue Y.J., Lou M.Q., Zhao W. (2017). Activation of DRD5 (Dopamine Receptor D5) Inhibits Tumor Growth by Autophagic Cell Death. Autophagy.

[93] Gu W., Zhang W., Wu Z., Cai Y. (2025). Cabergoline-Induced NDFIP1 Upregulation in Pituitary Neuroendocrine Tumor Cells Activates mTOR Signaling and Contributes to Cabergoline Resistance. J. Neuro-Oncol..

[94] Mauthe M., Orhon I., Rocchi C., Zhou X., Luhr M., Hijlkema K.J., Coppes R.P., Engedal N., Mari M., Reggiori F. (2018). Chloroquine Inhibits Autophagic Flux by Decreasing Autophagosome-Lysosome Fusion. Autophagy.

[95] Xu R., Ji Z., Xu C., Zhu J. (2018). The Clinical Value of Using Chloroquine or Hydroxychloroquine as Autophagy Inhibitors in the Treatment of Cancers: A Systematic Review and Meta-Analysis. Medicine.

[96] Shi T.T., Yu X.X., Yan L.J., Xiao H.T. (2017). Research Progress of Hydroxychloroquine and Autophagy Inhibitors on Cancer. Cancer Chemother. Pharmacol..

[97] Lin S.J., Wu Z.R., Cao L., Zhang Y., Leng Z.G., Guo Y.H., Shang H.B., Zhao W.G., Zhang X., Wu Z.B. (2017). Pituitary Tumor Suppression by Combination of Cabergoline and Chloroquine. J. Clin. Endocrinol. Metab..

[98] Lin S., Han C., Lou X., Wu Z.B. (2022). Hydroxychloroquine Overcomes Cabergoline Resistance in a Patient with Lactotroph Pituitary Neuroendocrine Tumor: A Case Report. Front. Endocrinol..

[99] ClinicalTrials.gov Cabergoline Combined Hydroxychloroquine/Chloroquine to Treat Resistant Prolactinomas (NCT03400865). NCT03400865.

[100] Nishioka H. (2023). Aggressive Pituitary Tumors (PitNETs). Endocr. J..

[101] Zhang K., Yang Y., Wang D., Xie B., Li X., Xu B. (2016). HIF-1α Inhibition Sensitized Pituitary Adenoma Cells to Temozolomide by Regulating Presenilin 1 Expression and Autophagy. Technol. Cancer Res. Treat..

[102] Cai L., Wu Z.R., Cao L., Xu J.D., Lu J.L., Wang C.D., Jin J.H., Wu Z.B., Su Z.P. (2022). ACT001 Inhibits Pituitary Tumor Growth by Inducing Autophagic Cell Death via MEK4/MAPK Pathway. Acta Pharmacol. Sin..

[103] Zhu J., Tang C., Cong Z., Yuan F., Cai X., Yang J., Ma C. (2021). ACT001 Reverses Resistance of Prolactinomas via AMPK-Mediated EGR1 and mTOR Pathways. Endocr.-Relat. Cancer.

[104] Li Z., Chen L., Zhang D., Huang X., Yang J., Li W., Wang C., Meng X., Huang G. (2023). Intranasal 15d-PGJ2 Inhibits the Growth of Rat Lactotroph Pituitary Neuroendocrine Tumors by Inducing PPARγ-Dependent Apoptotic and Autophagic Cell Death. Front. Neurosci..

[105] Sun H., Hu B., Wu C., Jiang T. (2024). Targeting the SPHK1/S1P/S1PR2 Axis Ameliorates GH-Secreted Pituitary Adenoma Progression. Eur. J. Clin. Investig..

[106] Zhang Y., Wang M., Ji C., Chen Z., Yang H., Wang L., Yu Y., Qiao N., Ma Z., Ye Z. (2021). Treatment of Acromegaly by Rosiglitazone via Upregulating 15-PGDH in Both Pituitary Adenoma and Liver. iScience.

[107] Du Q., Chen Z., Feng Z., Li Y., Xu L., Lin Y., Wang Y., Peng A., Feng Q. (2025). Valproic Acid Promotes Transcriptional Activation of Drd2 by Mediating Histone Acetylation to Inhibit the mTOR-Pttg1 Signaling Axis and Exerts Anti-PitNETs Activity. Phytomedicine.

[108] Wang Y., Pang B., Zhang R., Fu Y., Pang Q. (2019). Ubenimex Induces Apoptotic and Autophagic Cell Death in Rat GH3 and MMQ Cells Through the ROS/ERK Pathway. Drug Des. Dev. Ther..

[109] Cai Z., Qian B., Pang J., Tan Z.B., Zhao K., Lei T. (2022). Celastrol Induces Apoptosis and Autophagy via the AKT/mTOR Signaling Pathway in the Pituitary ACTH-Secreting Adenoma Cells. Curr. Med. Sci..

[110] Feng Y., Wang H., Zhou H., Li N., Liu Y., Lu Y., Wu Z.B., Ni D., Xue L. (2025). Targeting the Mitochondrial-Autophagy Axis in Pituitary Neuroendocrine Tumors via Intracellular Copper Overload. Chem. Eng. J..

[111] Yan Z., Liang C., Nian F., Peng G., Li Y., Guan J., Pan R., Song G. (2025). Curcumin for the Treatment of Pituitary Adenomas: The Potential of a Single Agent for Multifaceted Therapeutic Effects. Front. Endocrinol..

[112] Peshin S., Modi S., Babakhanlou R., Arshad J. (2025). Current Clinical Trial Landscape of Gastroenteropancreatic Neuroendocrine Tumors: A New Era of Landmark Trials. J. Clin. Med..

